# Addendum: Unified framework for open quantum dynamics with memory

**DOI:** 10.1038/s41467-025-61825-8

**Published:** 2025-08-12

**Authors:** Ruojing Peng, Felix Ivander, Lachlan P. Lindoy, Joonho Lee

**Affiliations:** 1 Department of Chemistry and Chemical Biology, Harvard University, Cambridge, MA 02138 USA; 2 Quantum Science and Engineering, Harvard University, Cambridge, MA USA; 3 National Physical Laboratory, Teddington, TW11 0LW United Kingdom

## Abstract

This Addendum presents a detailed analysis of the discretization error in time-integration and time-derivative that appear in the Nakajima-Zwanzig equation. This was brought to our attention by Makri et al. [arXiv:2410.08239]. Our analysis in the Addendum shows that the relationship derived in our earlier work [Nat. Commun. 15, 8087 (2024)] is valid within the choice of discretization and is not contaminated by the discretization error.

**Addendum to**: *Nature Communications* 10.1038/s41467-024-52081-3, published online 15 September 2024.

A recent commentary piece^1^ by Makri et al. raised a concern on the discretization error of the discrete-time Nakajima-Zwanzig (NZ) equation employed in our recent work^2^. In light of this, this Addendum provides a detailed analysis on the discretization error in the NZ equation and whether it affects our original analysis.

The continuous-time homogeneous NZ equation^3–5^ for the system propagator *U*(*t*) is

1 $$\dot{U}(t)=-i{L}_{s}U(t)+\mathop{\int}\nolimits_{0}^{t}d\tau {\mathcal{K}}(\tau )U(t-\tau )$$

where *L*_*s*_ = [*H*_*s*_, ⋅ ] and *U*(*t*) is defined by *ρ*(*t*) = *U*(*t*)*ρ*(0). For the subsequent analysis, it is useful to write its second-order derivative as derived in ref. ^6^ (see Supplementary Note I for detail)

2 $$\ddot{U}(t)={(-i{L}_{s})}^{2}U(t)+{\mathcal{K}}(t)+\mathop{\int}\nolimits_{0}^{t}d\tau {\mathcal{F}}(\tau )U(t-\tau )$$

where

3 $${\mathcal{F}}(t)=\{{\mathcal{K}}(t),-i{L}_{s}\}+\mathop{\int}\nolimits_{0}^{t}d\tau {\mathcal{K}}(\tau ){\mathcal{K}}(t-\tau ),$$

and the third-order derivative (see Supplementary Note II for detail)

4 $$\dddot{U}(t)=	 \left[{(-i{L}_{s})}^{3}+\{{{\mathcal{K}}}_{0},-i{L}_{s}\}+{\dot{{\mathcal{K}}}}_{0}\right]U(t)\\ 	 +\mathop{\int}\nolimits_{0}^{t}d\tau {\mathcal{R}}(t-\tau )U(\tau )$$

where

5 $${\mathcal{R}}(t)=\left[\left[{(-i{L}_{s})}^{2}+{{\mathcal{K}}}_{0}\right]{\mathcal{K}}-i{L}_{s}\dot{{\mathcal{K}}}+\ddot{{\mathcal{K}}}\right](t).$$

We note that ${\mathcal{F}}(t) \sim {\mathcal{O}}(| | {\mathcal{K}}(t)| | )$ (∣∣ ⋅ ∣∣ denotes Frobenius norm of the matrix) from Young’s convolution inequality^7,8^. Furthermore, $\dot{{\mathcal{K}}}(t)$, $\ddot{{\mathcal{K}}}(t)$, and hence ${\mathcal{R}}(t)$ are also ${\mathcal{O}}(| | {\mathcal{K}}(t)| | )$ from the projection of full system-and-bath evolution^9,10^.

We now consider the discrete-time version of Eqs. (1), (2) and (4). Following the same convention as our original manuscript, let *t* = *N*Δ*t*, *L* = 1 − *i*Δ*tL*_*s*_, *ρ*_*N*_ = *ρ*(*N*Δ*t*), *U*_*N*_ = *U*(*N*Δ*t*), ${{\mathcal{K}}}_{m}={\mathcal{K}}(m\Delta t)$ be the continuous-time memory kernel in Eq. (1) evaluated at discrete time steps, and the discrete-time memory kernel, *K*_*m*_, be defined by discrete time relations

6 $${U}_{N+1}=(I-i\Delta t{L}_{s}){U}_{N}+\Delta {t}^{2}\mathop{\sum }\limits_{m=0}^{N}{K}_{N-m}{U}_{m},$$

from ref. ^2^ and the discrete-time memory kernel, {*K*_*m*_}, is the central quantity used in our original article. Our goal is to demonstrate how the discretization error in the discrete NZ equation propagates into the relationship between ${{\mathcal{K}}}_{m}$ and *K*_*m*_ and whether the time-translational invariance of *K*_*m*_ is, in fact, incorrectly assumed and contaminated by the discretization error.

We begin by considering the time-derivatives of the system propagator at *t* = 0 (i.e., *N* = 0),

7 $${\ddot{U}}_{0}={(-i{L}_{s})}^{2}+{{\mathcal{K}}}_{0},$$

8 $${\dddot{U}}_{0}={(-i{L}_{s})}^{3}+\{{{\mathcal{K}}}_{0},-i{L}_{s}\}+{\dot{{\mathcal{K}}}}_{0}.$$

The expansion of *U*_1_ at *t* = 0 follows

9 $${U}_{1}=I+\Delta t {\dot{U}_{0}}+\frac{\Delta {t}^{2}}{2}{\ddot{U}}_{0}+\frac{\Delta {t}^{3}}{6}\;\;\;{\dddot{U}}_{0}+{\mathcal{O}}(\Delta {t}^{4}).$$

We can use Eqs. (9) and (6) to obtain

10 $${K}_{0}=\frac{1}{2}\left({(-i{L}_{s})}^{2}+{{\mathcal{K}}}_{0}\right)+\frac{\Delta t}{6}\;\;\;{\dddot{U}}_{0}+{\mathcal{O}}(\Delta {t}^{2}).$$

This reveals that our *K*_0_ is related to ${{\mathcal{K}}}_{0}$ up to an additive error ${\mathcal{O}}(\Delta t)$.

For *N* > 0, we discretize the time-convolution integral in Eqs. (2) and (4) with the left Riemann sum,

11 $${\ddot{U}}_{N}=	 {(-i{L}_{s})}^{2}{U}_{N}+{{\mathcal{K}}}_{N}+\Delta t\mathop{\sum }\limits_{m=0}^{N-1}{{\mathcal{F}}}_{N-m}{U}_{m}\\ 	 +{\mathcal{O}}(N\Delta {t}^{2}),$$

12 $${\dddot{U}}_{N}=\;\;\;{\dddot{U}}_{0}{U}_{N}+\Delta t\mathop{\sum }\limits_{m=0}^{N-1}{{\mathcal{R}}}_{N-m}{U}_{m}+{\mathcal{O}}(N\Delta {t}^{2}).$$

Next, the integral in Eq. (1) is approximated by the trapezoidal rule,

13 $${\dot{U}}_{N}=	 -i{L}_{s}{U}_{N}+\Delta t\left[\frac{1}{2}{{\mathcal{K}}}_{N}+\mathop{\sum }\limits_{m=1}^{N-1}{{\mathcal{K}}}_{N-m}{U}_{m}+\frac{1}{2}{{\mathcal{K}}}_{0}{U}_{N}\right]\\ 	+{\mathcal{O}}(N\Delta {t}^{3}).$$

Similarly to Eq. (9), the expansion of *U*_*N*+1_ at t = *N*Δ*t* follows (see Supplementary Note III for detail)

14 $${U}_{N+1}=	 {U}_{N}+\Delta t{\dot{U}}_{N}+\frac{\Delta {t}^{2}}{2}{\ddot{U}}_{N}+\frac{\Delta {t}^{3}}{6}\;\;\;{\dddot{U}}_{N}+{\mathcal{O}}(\Delta {t}^{4})\\=	 (I-i\Delta t{L}_{S}){U}_{N}+\Delta {t}^{2}\left[\mathop{\sum }\limits_{m=0}^{N-1}{{\mathcal{K}}}_{N-m}{U}_{m}+{K}_{0}{U}_{N}\right]\\ 	 +\frac{\Delta {t}^{3}}{2}\mathop{\sum }\limits_{m=0}^{N-1}{{\mathcal{F}}}_{N-m}{U}_{m}+{\mathcal{O}}(N\Delta {t}^{4})\\ 	+\frac{\Delta {t}^{4}}{6}\mathop{\sum }\limits_{m=0}^{N-1}{{\mathcal{R}}}_{N-m}{U}_{m}+{\mathcal{O}}(N\Delta {t}^{5}).$$

Finally, by comparing Eqs. (6) and (14), we observe

15 $${K}_{N}={{\mathcal{K}}}_{N}+\frac{\Delta t}{2}{{\mathcal{F}}}_{N}+{\mathcal{O}}(\Delta {t}^{2}),$$

which reveals how the discretization error used in Eq. (6) propagates into the discrete-time memory kernel. From Eqs. (10) and (15), we see that the discrete-time memory kernel *K*_*N*_ agrees with the continuous-time memory kernel ${{\mathcal{K}}}_{N}$ up to a discretization error of ${\mathcal{O}}(\Delta t)$ for *N* > 0, and they are related up to an additive error of ${\mathcal{O}}(\Delta t)$ by *L*_*s*_ at *N* = 0. Furthermore, the discrete-time memory kernel obeys time-translational invariance up to ${\mathcal{O}}(\Delta {t}^{2})$ since ${{\mathcal{F}}}_{N}$ is time-translationally invariant. Hence, our analysis in the original manuscript is valid up to the discretization error ${\mathcal{O}}(\Delta t)$, and the discretization error does not change our analysis. We also note that Cao et al. numerically demonstrated the convergence of *K*_*N*_ to ${{\mathcal{K}}}_{N}$ when taking the limit of Δ*t* → 0 in ref. ^11^, which is consistent with our analysis.

Our original work presented a formal, explicit one-to-one mapping between memory kernel and influence functions for various open-quantum system settings including spin, fermionic, and bosonic baths with commuting/non-commuting or diagonalizable/non-diagonalizable system-bath coupling. As mentioned in our original work as well as its supplementary materials and also in ref. ^1^, there are similarities among our analysis, Cao’s transfer tensor method^11^, and Makri’s small matrix method^12^, especially for the simplest setting of a bosonic bath with commuting, diagonalizable system-bath coupling. Our work presents a unified framework for open quantum dynamics for various settings beyond the available analyses and a quantum sensing protocol for a system coupled to Gaussian baths (i.e., learning spectral density from reduced system dynamics).

## Supplementary information

Supplementary Notes

## Data Availability

Data generated in this study is fully presented in the main text and Appendices.

## Code availability

No code has been used to generate the data.

## References

[1] Makri, N., Kundu, S., Cai, Z., and Wang, G. Comment on “unified framework for open quantum dynamics with memory”. Preprint at https://arxiv.org/abs/2410.08239 (2025).

[2] Ivander, F., Lindoy, L. P. & Lee, J. Unified framework for open quantum dynamics with memory. *Nat. Commun.* **15**, 8087 (2024). 39278965 10.1038/s41467-024-52081-3 PMC11402990

[3] Nakajima, S. On quantum theory of transport phenomena: Steady diffusion. *Prog. Theor. Phys.* **20**, 948 (1958).

[4] Zwanzig, R. Ensemble method in the theory of irreversibility. *J. Chem. Phys.* **33**, 1338 (1960).

[5] Mulvihill, E. & Geva, E. A road map to various pathways for calculating the memory kernel of the generalized quantum master equation. *J. Phys. Chem. B* **125**, 9834 (2021). 34424700 10.1021/acs.jpcb.1c05719

[6] Kidon, L., Wang, H., Thoss, M. & Rabani, E. On the memory kernel and the reduced system propagator. *J. Chem. Phys.* **149**, 104105 (2018). 30219023 10.1063/1.5047446

[7] Young, W. H. On the multiplication of successions of fourier constants. *Proc. R. Soc. Lond. Ser. A, Containing Pap. A Math. Phys. Character* **87**, 331 (1912).

[8] Bogachev, V. I., *Measure Theory*, 2007th ed., Vol. 1 (Springer, Berlin, Germany, 2006).

[9] Shi, Q. & Geva, E. A new approach to calculating the memory kernel of the generalized quantum master equation for an arbitrary system-bath coupling. *J. Chem. Phys.* **119**, 12063 (2003). 10.1063/1.1738109 15268091

[10] Zhang, M.-L., Ka, B. J. & Geva, E. Nonequilibrium quantum dynamics in the condensed phase via the generalized quantum master equation. *J. Chem. Phys.* **125**, 044106 (2006). 10.1063/1.2218342 16942133

[11] Cerrillo, J. & Cao, J. Non-markovian dynamical maps: Numerical processing of open quantum trajectories. *Phys. Rev. Lett.* **112**, 110401 (2014). 24702332 10.1103/PhysRevLett.112.110401

[12] Makri, N. Small matrix path integral for system-bath dynamics. *J. Chem. Theory Comput.* **16**, 4038 (2020). 32538630 10.1021/acs.jctc.0c00039

