# Supplementary material for: Unified framework for open quantum dynamics with memory
Source: Nat Commun. 2024 Sep 15;15:8087. doi: 10.1038/s41467-024-52081-3 (PMC11402990; doi:10.1038/s41467-024-52081-3)
Supplement: Supplementary file 1 — Supplementary Information [file 41467_2024_52081_MOESM1_ESM.pdf]

Supplementary Notes for: Unified Framework for Open Quantum Dynamics with Memory

Felix Ivander,[1] Lachlan P. Lindoy,[2] and Joonho Lee[3,4,*]

[1] *Quantum Science and Engineering, Harvard University, Cambridge, MA, USA*
[2] *National Physical Laboratory, Teddington, TW11 0LW, United Kingdom*
[3] *Department of Chemistry and Chemical Biology, Harvard University, Cambridge, MA, USA*
[4] *Google Quantum AI, Venice, CA, USA*

Supplementary Note 1. PATH INTEGRAL FORMULATION FOR GAUSSIAN BATHS

We present a more detailed formulation of the influence-functional-based path-integral (INFPI) approaches.

A. General formulation

We consider the general Hamiltonian of a quantum system interacting with external degrees of freedom,

$$\hat{H} = \hat{H}_S + \sum_j \left(\hat{H}_{B,j} + \sum_\alpha \hat{H}_{I,j,\alpha} \right), \tag{1}$$

where $\hat{H}_S$ is the system Hamiltonian, $\hat{H}_{B,j} = \sum_k \omega_{k,j} \hat{a}^\dagger_{k,j} \hat{a}_{k,j}$, and $\hat{H}_{I,j,\alpha} = \hat{S}_{j,\alpha} \otimes \hat{B}_{j,\alpha}$ where $\hat{S}_{j,\alpha}$ is a system operator and $\hat{B}_{j,\alpha}$ is a bath operator for the α-th interaction term. The time evolution of the density matrix of the full system, $\rho_{\text{tot}}(t)$, follows

$$\rho_{\text{tot}}(t) = e^{-i\hat{H}t} \rho_{\text{tot}}(0) e^{i\hat{H}t}. \tag{2}$$

In INFPI, the real-time propagator is evaluated by path-integral.

With the quasiadiabatic approximation, one may split the total Hamiltonian as (here $\hat{H}_{\text{env}} = \hat{H} - \hat{H}_S = \sum_j \hat{H}_{B,j} + \sum_\alpha \hat{H}_{I,j,\alpha}$)

$$e^{-i\hat{H}\Delta t} \simeq e^{-i\hat{H}_S \Delta t/2} e^{-i\hat{H}_{\text{env}}\Delta t} e^{-i\hat{H}_S \Delta t/2} + O(\Delta t^3). \tag{3}$$

With this identity, we express the trotterized evolution as (a summation over the paths implied)

$$\begin{aligned} \langle x^+_{2N} | \rho_{\text{tot}}(t) | x^-_{2N} \rangle &= \langle x^+_{2N} | e^{-i\hat{H}_S \Delta t/2} e^{-i\hat{H}_{\text{env}}\Delta t} e^{-i\hat{H}_S \Delta t/2} | x^+_{2N-2} \rangle \\ &\quad \cdots \langle x^+_0 | \rho_{\text{tot}}(0) | x^-_0 \rangle \cdots \langle x^-_{2N-2} | e^{i\hat{H}_S \Delta t/2} e^{i\hat{H}_{\text{env}}\Delta t} e^{i\hat{H}_S \Delta t/2} | x^-_{2N} \rangle. \end{aligned} \tag{4}$$

We further insert resolution-of-the-identities between every $e^{\pm i\hat{H}_S \Delta t/2}$ and $e^{i\hat{H}_{\text{env}}\Delta t}$ to obtain

$$\langle x^+_{2N} | \rho_{\text{tot}}(t) | x^-_{2N} \rangle = \langle x^+_{2N} | e^{-i\hat{H}_S \Delta t/2} | x^{+'}_{2N} \rangle \langle x^{+'}_{2N} | e^{-i\hat{H}_{\text{env}}\Delta t} | x^{+'}_{2N-2} \rangle \langle x^{+'}_{2N-2} | e^{-i\hat{H}_S \Delta t/2} | x^+_{2N-2} \rangle \cdots . \tag{5}$$

We consider a product-separable initial condition,

$$\rho_{\text{tot}}(0) = \rho(0) \otimes \left[\frac{\exp\left[-\beta_j \hat{H}_{B,j}\right]}{\text{Tr}_B\left(\exp\left[-\beta_j \hat{H}_{B,j}\right]\right)} \right]^{\otimes j} = \rho(0) \otimes (\rho_{B,eq,j})^{\otimes j}. \tag{6}$$

With this, upon tracing out the bath degrees of freedom, we have

$$\langle x^+_{2N} | \rho(t) | x^-_{2N} \rangle = G_{x^\pm_{2N} x^{\pm'}_{2N}} G_{x^{\pm'}_{2N} x^\pm_{2N-1}} \cdots \langle x^+_0 | \rho(0) | x^-_0 \rangle \, \text{Tr}_B[\langle x^+_{2N} | e^{-i\hat{H}_{env}\Delta t} | x^{+'}_{2N} \rangle \cdots \rho_{B,eq} \cdots \langle x^{-'}_{2N} | e^{-i\hat{H}_{env}\Delta t} | x^-_{2N} \rangle] \tag{7}$$

where $\rho(t) = \text{Tr}_B(\rho_{\text{tot}}(t))$ and $G_{x^\pm y^\pm} = \langle x^+ | e^{-i\hat{H}_s \Delta t/2} | y^+ \rangle \langle y^- | e^{i\hat{H}_s \Delta t/2} | x^- \rangle$. The term with the trace over bath degrees of freedom in Section Supplementary Note 1 A is the so-called *influence functional* (IF). Up to this point, no assumptions have been made on the form of $\hat{S}_{j,\alpha}$, $\hat{B}_{j,\alpha}$, and the algebra of $\hat{a}_{k,j}$.

B. Influence Functionals for independent Gaussian Baths

The evaluation of the IF for general interacting baths involves complicated numerical procedures. However, one can make more progress if baths follow Gaussian statistics, are all independent from each other, and correspond to only one interaction coupling term.

In such cases, the calculation of the influence functional for Gaussian baths have been presented elsewhere[1–3], but we sketch here briefly for completeness. With the Hamiltonian Eq. (1), the expression for the dynamics of the total density matrix is expressed as, in Liouville space in the interaction picture,

$$\frac{d}{dt}\rho_{\rm tot}(t) = \sum_j \mathcal{L}_{I,j}(t)\rho_{\rm tot}(t) \tag{8}$$

which is solved by

$$\rho_{\rm tot}(t) = \mathcal{T}\prod_j e^{\int_0^t \mathcal{L}_{I,j}(\tau)d\tau}\rho_{\rm tot}(0), \tag{9}$$

where the product form is due to the commuting property of integrals under time-ordering[4]. There, $\mathcal{T}$ denotes the time-ordering operator, and $\mathcal{L}_{I,j}$ is the interaction Hamiltonian (with respect to H_S and $H_{B,j}$) in Liouville space, $\mathcal{L}_{I,j}\bullet = -i[H_{I,j}(t),\bullet]$ (hats omitted). Now, taking the trace over multiple environments, we have

$$\rho(t) = \mathrm{Tr}_{B,N}[\ldots\mathrm{Tr}_{B,2}[\mathrm{Tr}_{B,1}[\rho_{\rm tot}(t)]]\ldots] = \prod_j \langle \mathcal{T}e^{\int_0^t \mathcal{L}_{I,j}(\tau)d\tau}\rangle_j \rho(0), \tag{10}$$

where $\langle\cdot\rangle_j$ denotes taking the average over the environment j. The averaged quantity above is precisely the continuous IF. Now, as the trace over each bath separates into an independent term in the product above, we will consider evaluating one term and, in what follows, will drop the subscript j. Applying Kubo's generalized cumulant identity[5] yields

$$\langle \mathcal{T}e^{\int_0^t \mathcal{L}_I(\tau)d\tau}\rangle = \sum_{n=0}^{\infty}\frac{1}{n!}\langle\mathcal{T}\int_0^t d\tau_1\cdots\int_0^t d\tau_n \mathcal{L}_I(\tau_1)\ldots\mathcal{L}_I(\tau_n)\rangle. \tag{11}$$

Then, an application of Isserlis' Theorem[6] (a special case of the well-known Wick's Theorem[7]), which states that if the random variables X_j have zero mean and are normal (i.e., Gaussian statistics) then $\langle X_1\ldots X_n\rangle = \sum_{p\in P_n^2}\prod_{\{i,j\}\in p}\langle X_iX_j\rangle$ where the sum run overs all possible pairings of $\{1,\ldots,n\}$ and the product over the pairs in p, yields

$$\begin{aligned}\langle\mathcal{T}\int_0^t d\tau_1\cdots\int_0^t d\tau_n \mathcal{L}_I(\tau_1)\ldots\mathcal{L}_I(\tau_n)\rangle &= \sum_{p\in P_n^2}\mathcal{T}\int_0^t d\tau_i\int_0^t d\tau_j\prod_{\{i,j\}\in p}\langle\mathcal{L}_I(\tau_i)\mathcal{L}_I(\tau_j)\rangle \\ &= \mathcal{T}\frac{2n!}{(2^n n!)}\Big(\int_0^t d\tau_i\int_0^t d\tau_j\langle\mathcal{L}_I(\tau_i)\mathcal{L}_I(\tau_j)\rangle\Big)^n.\end{aligned} \tag{12}$$

Since the odd moments vanish, there we skipped the odd terms (there are $\frac{(2n)!}{(2^n n!)}$ such pair partitions). Furthermore, because of this property, when we reinsert to Eq. (11) we obtain

$$\begin{aligned}\langle\mathcal{T}e^{\int_0^t \mathcal{L}_I(\tau)d\tau}\rangle &= \sum_{n=0}^{\infty}\frac{1}{(2n)!}\mathcal{T}\frac{2n!}{(2^n n!)}\Big(\int_0^t d\tau_i\int_0^t d\tau_j\langle\mathcal{L}_I(\tau_i)\mathcal{L}_I(\tau_j)\rangle\Big)^n \\ &= \sum_{n=0}^{\infty}\frac{1}{n!}\Big(\int_0^t d\tau_i\int_0^t d\tau_j\frac{\langle\mathcal{T}\mathcal{L}_I(\tau_i)\mathcal{L}_I(\tau_j)\rangle}{2}\Big)^n.\end{aligned} \tag{13}$$

Then, applying Fubini's theorem[8] as well as recalling that the above is the Taylor expansion of an exponential we finally get

$$\langle\mathcal{T}e^{\int_0^t \mathcal{L}_I(\tau)d\tau}\rangle = e^{\int_0^t d\tau\int_0^\tau d\tau'\langle\mathcal{T}\mathcal{L}_I(\tau)\mathcal{L}_I(\tau')\rangle} \tag{14}$$

which is the continuous version of the influence functional in the main work. Another way to see this result is to use $\langle e^X\rangle = e^{\langle X\rangle^2/2}$ if X is Gaussian. Note, when moving beyond Gaussian environments, one would need to consider

each term beyond the second order in the cumulant expansion of Eq. (11) as Isserlis' Theorem becomes inapplicable. Thus, the inversion procedure we showed in the main text could be seen as approximating the action of an anharmonic environment by that of a harmonic one.

For a given open-quantum system Hamiltonian, one can write a specific form for Eq. (14) and evaluate the IF via the discrete path-integral approach. Once the INFPI expression is discretized, a matrix tensor, **I**, known as the influence function, appears. They are closely related to the bath-bath correlation functions. We will show the computation of **I** from microscopic parameters. We must consider each class through different models as the form of bath-bath correlation functions will slightly differ. We summarize the differences between the classes considered in Table 1.

	Diagonalizable $\{\hat{S}_{j,\alpha}\}$	Simultaneously diagonalizable $\{\hat{S}_{j,\alpha}\}$	Single $\{\hat{H}_{I,j,\alpha}\}$ for each bath
Class 1	✓	✓	✓
Class 2	✓	✗	✓
Class 3	✓	✗	✗
Class 4	✗	✗	✗

Supplementary Table 1. Summary of different *Classes* considered in this work.

Supplementary Note 2. THE NAKAJIMA-ZWANZIG EQUATION

The Nakajima-Zwanzig (NZ) equation takes the form[9]

$$\dot{\rho}(t) = -\frac{i}{\hbar}\mathcal{L}\rho(t) + \int_0^t d\tau \boldsymbol{\mathcal{K}}(t,\tau)\rho(\tau) + \mathcal{I}(t). \tag{15}$$

In this work, the inhomogeneous term $\mathcal{I}(t)$ vanishes due to the factorized initial condition assumption $\rho_{\text{tot}}(0) = \rho(0) \otimes \rho_{B,j}^{\otimes j}$. The *discretized* form of the homogeneous NZ equation is (with $t = (N-1)\Delta t$)

$$\frac{\rho_N - \rho_{N-1}}{\Delta t} = -\frac{i}{\hbar}\mathcal{L}_s\rho_{N-1} + \sum_{m=1}^{N} \boldsymbol{\mathcal{K}}_{N,m}\rho_m\Delta t. \tag{16}$$

By noting that $\rho_N = U_N\rho_0$ (where $\rho_N = \rho(N\Delta t)$) we get

$$\mathbf{U}_N = \mathbf{L}\mathbf{U}_{N-1} + \Delta t^2 \sum_{m=1}^{N} \boldsymbol{\mathcal{K}}_{N,m}\mathbf{U}_{m-1}. \tag{17}$$

This will allow obtaining $\boldsymbol{\mathcal{K}}$ in terms of $\mathbf{I}$ by making substitutions to $\mathbf{U}_k$.

First, we organize equations expressing $\mathbf{U}_N$ in terms of $\boldsymbol{\mathcal{K}}_{N,m}$ as follows, for $N = 1$,

$$\frac{\rho_1 - \rho_0}{\Delta t} = -\frac{i}{\hbar}\mathcal{L}_s\rho_0 + \boldsymbol{\mathcal{K}}_{1,1}\rho_0\Delta t \tag{18}$$

so

$$\mathbf{U}_1 = (\mathbf{I} - \frac{i}{\hbar}\mathcal{L}_s\Delta t) + \boldsymbol{\mathcal{K}}_{1,1}\Delta t^2. \tag{19}$$

For $N = 2$,

$$\rho_2 = (\mathbf{I} - \frac{i}{\hbar}\mathcal{L}_s\Delta t)\rho_1 + [\boldsymbol{\mathcal{K}}_{2,1}\rho_0 + \boldsymbol{\mathcal{K}}_{2,2}\rho_1]\Delta t^2. \tag{20}$$

Similarly, for $N = 3$,

$$\rho_3 = (\mathbf{I} - \frac{i}{\hbar}\mathcal{L}_s\Delta t)\rho_2 + [\boldsymbol{\mathcal{K}}_{3,1}\rho_0 + \boldsymbol{\mathcal{K}}_{3,2}\rho_1 + \boldsymbol{\mathcal{K}}_{3,3}\rho_2]\Delta t^2. \tag{21}$$

Note that so far Eq. 19 to 21 are exact and general.

Inspecting Eq. (20), one observes that with the time-translational invariance of the memory kernels (i.e., valid if the system Hamiltonian does not have explicit time-dependence),

$$\boldsymbol{\mathcal{K}}_{2,1} = \boldsymbol{\mathcal{K}}_{3,2} \equiv \boldsymbol{\mathcal{K}}_1. \tag{22}$$

We use this and make appropriate substitutions in our previous results. Then, we obtain

$$\boldsymbol{\mathcal{K}}_1 \rho_0 = \frac{1}{\Delta t^2}\Big[\mathbf{U}_2 - \mathbf{U}_1\mathbf{U}_1\Big]\rho_0, \tag{23}$$

or

$$\boldsymbol{\mathcal{K}}_1 = \frac{1}{\Delta t^2}(\mathbf{U}_2 - \mathbf{U}_1\mathbf{U}_1) \tag{24}$$

Then, for Eq. (21) we reorganize to

$$\begin{aligned}\mathbf{U}_3\rho_0 &= (\mathbf{1} - \frac{i}{\hbar}\mathcal{L}_s\Delta t)\mathbf{U}_2\rho_0 + [\boldsymbol{\mathcal{K}}_{3,1} + \frac{1}{\Delta t^2}(\mathbf{U}_2 - \mathbf{U}_1\mathbf{U}_1)\mathbf{U}_1 \\ &\quad + \frac{1}{\Delta t^2}(\frac{i}{\hbar}\mathcal{L}_s\Delta t - 1 + \mathbf{U}_1)\mathbf{U}_2\rho_0\Delta t^2. \end{aligned}\tag{25}$$

One observes many terms again cancel, leaving

$$\boldsymbol{\mathcal{K}}_2 = \frac{1}{\Delta t^2}\Big[-\mathbf{U}_2\mathbf{U}_1 + \mathbf{U}_1\mathbf{U}_1\mathbf{U}_1 - \mathbf{U}_1\mathbf{U}_2 + \mathbf{U}_3\Big]. \tag{26}$$

One can proceed in similar fashion for higher order terms. For $N = 4$,

$$\begin{aligned}\rho_4 &= (\mathbf{1} - \frac{i}{\hbar}\mathcal{L}_s\Delta t)\rho_3 + [\boldsymbol{\mathcal{K}}_{4,1}\rho_0 + \boldsymbol{\mathcal{K}}_{4,2}\rho_1 + \boldsymbol{\mathcal{K}}_{4,3}\rho_2 + \boldsymbol{\mathcal{K}}_{4,4}\rho_3]\Delta t^2 \\ \mathbf{U}_4\rho_0 &= (\mathbf{1} - \frac{i}{\hbar}\mathcal{L}_s\Delta t)\mathbf{U}_3\rho_0 + [\boldsymbol{\mathcal{K}}_{4,1} + \boldsymbol{\mathcal{K}}_2\mathbf{U}_1 + \frac{1}{\Delta t^2}(\mathbf{U}_2 - \mathbf{U}_1\mathbf{U}_1)\mathbf{U}_2 \\ &\quad + \frac{1}{\Delta t^2}(\frac{i}{\hbar}\mathcal{L}_s\Delta t - 1 - \mathbf{U}_1)\mathbf{U}_3]\rho_0\Delta t^2. \end{aligned}\tag{27}$$

This then gives

$$\boldsymbol{\mathcal{K}}_3 = \frac{1}{\Delta t^2}\Big[\mathbf{U}_4 + \mathbf{U}_2\mathbf{U}_1\mathbf{U}_1 - \mathbf{U}_1\mathbf{U}_1\mathbf{U}_1\mathbf{U}_1 + \mathbf{U}_1\mathbf{U}_2\mathbf{U}_1 - \mathbf{U}_3\mathbf{U}_1 - \mathbf{U}_2\mathbf{U}_2 + \mathbf{U}_1\mathbf{U}_1\mathbf{U}_2 - \mathbf{U}_1\mathbf{U}_3\Big]. \tag{28}$$

This series of equations will be the starting point to express $\boldsymbol{\mathcal{K}}$ in terms of $\mathbf{I}$ for different models that represent the different *Classes* discussed in the main text, in the following Supplementary Notes. We note that expressing $\mathbf{K}$ in terms of $\mathbf{U}$ was also noted in the transfer tensor method by Cerrillo and Cao,[10] but their approach does not illustrate how to write $\mathbf{K}$ in terms of $\mathbf{I}$ in the end. In the following subsections, we will show that one can write $\mathbf{U}$ in terms of $\mathbf{I}$ and therefore write $\mathbf{K}$ in terms of $\mathbf{I}$.

Supplementary Note 3. ADDITIONAL DETAILS ON *CLASS 1*

To make more progress, we assume that $\hat{S}_j$ is diagonalizable, all $\{\hat{S}_j\}$ are simultaneously diagonalizable. We later consider generalizations beyond these assumptions.

A. Influence functionals for *Class 1*

Under the assumptions of *Class 1*, the system coupling operators share the same eigenbasis. We thus insert the resolution-of-the-identity in this basis between the exponentials. The terms $\langle x_n|\Big(\prod_\alpha e^{-i\hat{H}_{\mathrm{env},\alpha}\Delta t}\Big)|x_n'\rangle$ are evaluated via successive application of the exponential operator to the ket

$$e^{-i\hat{H}_{\mathrm{env},\alpha}\Delta t/2}|x_n\rangle = e^{-iH_{\mathrm{env},\alpha}(x_n)\Delta t/2}|x_n\rangle, \tag{29}$$

and then we use $\langle x_n | x'_n \rangle = \delta_{x_n x'_n}$ to obtain

$$\langle x_{2N}^+ | \rho(t) | x_{2N}^- \rangle = G_{x_{2N}^\pm x_{2N-1}^\pm} G_{x_{2N-1}^\pm x_{2N-2}^\pm} \cdots \langle x_0^+ | \rho(0) | x_0^- \rangle \tag{30}$$

$$\times \prod_\alpha \mathrm{Tr}_{B,\alpha} \left[e^{-i\hat{H}_{\mathrm{env},\alpha}(x_{2N-1}^+)\Delta t} \cdots \rho_{B,eq} \cdots e^{i\hat{H}_{\mathrm{env},\alpha}(x_{2N-1}^-)\Delta t} \right] \tag{31}$$

Here, the influence functional is

$$\mathcal{I}(x_1^\pm, x_3^\pm, \cdots, x_{2N-1}^\pm) = \prod_\alpha \mathrm{Tr}_{B,\alpha} \left[e^{-i\hat{H}_{\mathrm{env},\alpha}(x_{2N-1}^+)\Delta t} \cdots \rho_{B,eq} \cdots e^{i\hat{H}_{\mathrm{env},\alpha}(x_{2N-1}^-)\Delta t} \right]. \tag{32}$$

We will see how this functional can be calculated for Gaussian baths.

B. Representative models in *Class 1*

1. *Gaussian Bosonic Environment*

Since generalizing the spin to multilevel systems will be straightforward, for clarity, we consider here the spin-boson model (details in the main text), described by $\hat{H}_S = \epsilon\sigma_z + \Delta\sigma_x$, coupled via σ_z to a harmonic bath with spectral density ($\omega \geq 0$) Eq. (17) in the main text. Note that although here we consider coupling only to a single environment, as we saw in Supplementary Note 1, generalizing to multiple additive environments is straightforward. The spin-boson is paradigmatic in the sense that many other models share its universality class, including the double-quantum-dot model and the low-temperature limit of dissipating molecules.[11] With the spin-boson Hamiltonian, we open the exponent integrand in Eq. (14),

$$\langle \mathcal{L}_I(\tau)\mathcal{L}_I(\tau') \rangle = -(x^+(\tau) - x^-(\tau))(\langle B(\tau)B(\tau') \rangle_B x^+(\tau') - \langle B(\tau)B(\tau') \rangle_B^* x^-(\tau')), \tag{33}$$

where $x^\pm \in$ pair$\{+1, -1\}$ in this case since the coupling is via σ_z. For the spin-boson model,

$$\begin{aligned} \langle B(\tau)B(\tau') \rangle_B &= \Big\langle \sum_j \lambda_j (\hat{b}_j^\dagger e^{i\omega_j t} + \hat{b}_j e^{-i\omega_j t}) \sum_k \lambda_k (\hat{b}_k^\dagger + \hat{b}_k) \Big\rangle \qquad (\tau - \tau' \to t) \\ &= \sum_{j,k} \lambda_j \lambda_k [\langle \hat{b}_j^\dagger e^{i\omega_j t} \hat{b}_k^\dagger \rangle + \langle \hat{b}_j^\dagger e^{i\omega_j t} \hat{b}_k \rangle + \langle \hat{b}_j e^{-i\omega_j t} \hat{b}_k^\dagger \rangle + \langle \hat{b}_j e^{-i\omega_j t} \hat{b}_k \rangle] \\ &= \sum_j \lambda_j^2 [\langle \hat{b}_j^\dagger e^{i\omega_j t} \hat{b}_j \rangle + \langle \hat{b}_j e^{-i\omega_j t} \hat{b}_j^\dagger \rangle] \\ &= \sum_j \lambda_j^2 [e^{i\omega_j t} \langle \hat{n}(\omega_j) \rangle + e^{-i\omega_j t} \langle \hat{n}(\omega_j) + 1 \rangle] \end{aligned} \tag{34}$$

We now take the continuous limit, and with $n(\omega) = \frac{1}{e^{\beta\omega}-1}$ so that $2n(\omega) + 1 = \frac{2}{e^{\beta\omega}-1} + 1 = \frac{e^{\beta\omega}+1}{e^{\beta\omega}-1} = \frac{e^{\beta\omega/2}+e^{-\beta\omega/2}}{e^{\beta\omega/2}-e^{-\beta\omega/2}}$ this expands to

$$\frac{1}{\pi} \int_0^\infty d\omega J(\omega) [\frac{e^{\beta\omega/2}}{e^{\beta\omega/2} - e^{-\beta\omega/2}} e^{-i\omega t} + \frac{e^{-\beta\omega/2}}{e^{\beta\omega/2} - e^{-\beta\omega/2}} e^{i\omega t}] \tag{35}$$

If we allow $J(\omega) = -J(-\omega)$, this integral becomes half of an even integrand. Thus

$$\langle B(t)B(0) \rangle_B = \frac{1}{2\pi} \int_{-\infty}^\infty d\omega J(\omega) \frac{e^{\beta\omega/2} e^{-i\omega t}}{\sinh \beta\omega/2}. \tag{36}$$

Now we start discretizing the path (with $t_N = N\Delta t$),

$$x^\pm(t) = x_0^\pm(t)\Theta(t - t_0) + \sum_{k=1}^N (x_k^\pm - x_{k-1}^\pm \Theta(t - t_k)) \tag{37}$$

and see that the IFs are pairwise decomposable (two integrals in the exponent become a double summation), yielding

$$I_{k'-k, x_k^\pm x_{k'}^\pm} = \exp\{-(x_k^+ - x_k^-)(\eta_{kk'} x_{k'}^+ - \eta_{kk'}^* x_{k'}^-)\}, \tag{38}$$

where we have also used the time-translational property of the IFs and thus defined them in terms of time differences only. There, the coefficients $\eta_{kk'}$ are obtained by substituting the discretized path Eq. (37) to Eq. (33)

$$\eta_{kk'} = \frac{2}{\pi}\int_{-\infty}^{\infty} d\omega\Big(\frac{J(\omega)}{\omega^2}\frac{\exp\{\beta\hbar\omega/2\}}{\sinh\beta\hbar\omega/2}\sin^2(\omega\Delta t/2)e^{-i\omega\Delta t(k-k')}\Big), \tag{39}$$

$$\eta_{kk} = \frac{1}{2\pi}\int_{-\infty}^{\infty} d\omega\frac{J(\omega)}{\omega^2}\frac{\exp\{\beta\hbar\omega/2\}}{\sinh\beta\hbar\omega/2}(1-e^{-i\omega\Delta t}). \tag{40}$$

We remark that generalizing to multilevel systems (while still coupled to the bosonic environment), for example, in Frenkel excitonic models, amounts only to letting $x(t)$ take more and different values.

2. *Spin-Fermion via Bosonization*

Here we consider a central system, $\hat{H}_S = \epsilon_0\sigma_z + \Delta\sigma_x$, interacting with a fermionic environment, $\hat{H}_F = \sum_k \omega_k \hat{c}_k^\dagger \hat{c}_k$ through the interaction Hamiltonian $\hat{H}_I = |1\rangle\langle 1|\frac{V}{L}\sum_{k,q} c_k^\dagger c_q$. In the low energy description, when the impurity potential does not create a bound state, this model can be recast to a spin-boson Hamiltonian[12]

$$\hat{H} = \big(\epsilon - \int_0^{E_F}\frac{dE}{\pi}\delta(\sqrt{2mE})\big)\hat{\sigma}_z + \Delta\hat{\sigma}_x + \sum_q v_F|q|\hat{b}_q^\dagger\hat{b}_q + V\hat{\sigma}_z\sum_{q>0}\sqrt{\frac{q}{2\pi L}}(\hat{b}_q^\dagger + \hat{b}_q). \tag{41}$$

We can thus analyze this model as though it is a spin-boson model. Particularly, the inverse mapping procedure would extract microscopic parameters here, e.g., V, since the spectral density is then given by

$$J(\omega) = \sum_q 4V^2\frac{q}{2\pi L}\delta(\omega - v_F|q|) \tag{42}$$

3. *Gaussian Fermionic Environment*

Here we consider a system coupled to a fermionic environment,

$$\hat{H} = \hat{H}_s + \sum_k \omega_k\hat{c}_k^\dagger\hat{c}_k + \hat{S}\sum_k g_k(\hat{c}_k^\dagger + \hat{c}_k). \tag{43}$$

where $\hat{c}_k$ ($\hat{c}_k^\dagger$) are the fermionic annihilation (creation) operators, $\{\hat{c}_j, \hat{c}_k^\dagger\} = \delta_{jk}$, and we require that $\hat{S}$ is diagonalizable. For the spin-fermion model, $\hat{H}_s = \epsilon_0\sigma_z + \Delta\sigma_x$ and $\hat{S} = \sigma_z$. Starting from Eq. (33), the bath correlation function is different. That is,

$$\begin{aligned}
\langle B(\tau)B(\tau')\rangle_B &= \Big\langle\sum_j g_j(\hat{c}_j^\dagger e^{i\omega_j(\tau-\tau')} + \hat{c}_j e^{-i\omega_j(\tau-\tau')})\sum_k g_k(\hat{c}_k^\dagger + \hat{c}_k)\Big\rangle \\
&= \sum_{jk}\langle g_j(\hat{c}_j^\dagger e^{i\omega_j(\tau-\tau')} + \hat{c}_j e^{-i\omega_j(\tau-\tau')})g_k(\hat{c}_k^\dagger + \hat{c}_k)\rangle \\
&= \sum_j g_j^2[\langle\hat{c}_j^\dagger e^{i\omega_j t}\hat{c}_j\rangle + \langle\hat{c}_j e^{-i\omega_j t}\hat{c}_j^\dagger\rangle] \\
&= \sum_j g_j^2[e^{i\omega_j t}\langle\hat{n}_F(\omega_j)\rangle + e^{-i\omega_j t}\langle 1 - \hat{n}_F(\omega_j)\rangle],
\end{aligned} \tag{44}$$

and taking the continuous limit in addition to using the spectral density $J(\omega) = \pi\sum_j \lambda_j^2\delta(\omega - \omega_j)$, we get

$$\frac{1}{\pi}\int_0^\infty d\omega J(\omega)[e^{i\omega_j t}(n_F(\omega_j)) + e^{-i\omega_j t}(1 - n_F(\omega_j))] = \frac{1}{\pi}\int_0^\infty d\omega J(\omega)[\cos(\omega t) + i\sin(\omega t)(2n_F(\omega) - 1)]. \tag{45}$$

Recall $n_F(\omega) = \frac{1}{e^{\beta(\omega-\mu)}+1}$ so $2n_F(\omega) - 1 = \frac{2}{e^{\beta(\omega-\mu)}+1} - 1 = \frac{e^{\beta(\omega-\mu)/2} - e^{-\beta(\omega-\mu)/2}}{e^{\beta(\omega-\mu)/2} + e^{-\beta(\omega-\mu)/2}}$. So, if we allow $J(\omega) = -J(-\omega)$, this integral becomes half of an even integrand. As a result

$$\langle B(t)B(0)\rangle_B = \frac{1}{2\pi}\int_{-\infty}^{\infty} d\omega J(\omega)\frac{e^{\beta(\omega-\mu)/2}e^{-i\omega t}}{\cosh\beta(\omega-\mu)/2}. \tag{46}$$

Conveniently, this expression Eq. (46)is only slightly modified compared to the analysis for the spin-boson model. Furthermore, the following relations. See Ref. 13 with which this result coincides.

4. Gaussian Spin Environment

Now we consider a system coupled with a diagonalizable coupling operator to a spin bath (again, the generalization to multiple such baths is straightforward if all the coupling operators commute). The Hamiltonian for this model reads[14–16]

$$\hat{H} = \hat{H}_s + \frac{1}{2}\sum_i^{N_s} \omega_i \hat{\sigma}_z^i + \hat{S}\frac{1}{2}\sum_i^{N_s} \frac{c_i}{\sqrt{2\omega_i}}\hat{\sigma}_x^i. \tag{47}$$

Note that environmental spins are non-interacting. Further, they are coupled to the central system in a manner by which $c_i \propto \frac{1}{\sqrt{N_s}}$ such that these contributions vanish in the thermodynamic limit–this restriction is physically motivated and common in the literature[14–16]. To follow we consider concretely the spin-spin bath model $\hat{H}_s = \epsilon\hat{\sigma}_z + \Delta\hat{\sigma}_x$ and $\hat{S} = \hat{\sigma}_z$. Even for this simplified model, although a Gaussian nature cannot in general be assumed[17] when considering the thermodynamic limit of the spins ($N_s \to \infty$), one obtains an effective spin-boson picture where the spectral density is renormalized[18]

$$J_{eff}(\omega, \beta) = \tanh \beta\omega/2J(\omega). \tag{48}$$

As a result, our results from previous models also apply to this case, mapping this problem to the spin-boson model.

C. System propagator in terms of influence functions

Returning from the end of Supplementary Note 3 A, going into Eq. (4) in the main text for Gaussian environments, if one writes the propagators in a more compact form (for $k > 0$)

$$\begin{aligned} U_{k,x_{2k}^{\pm}x_0^{\pm}} &= \sum_{x_{2k-1}^{\pm}} G_{x_{2k}^{\pm}x_{2k-1}^{\pm}} \sum_{x_1^{\pm}} \tilde{U}_{k-1,x_{2k-1}^{\pm}x_1^{\pm}} G_{x_1^{\pm}x_0^{\pm}}, \\ F_{x_{2k+1}^{\pm}x_{2k-1}^{\pm}} &= \sum_{x_{2k}^{\pm}} G_{x_{2k+1}^{\pm}x_{2k}^{\pm}} G_{x_{2k}^{\pm}x_{2k-1}^{\pm}}, \end{aligned} \tag{49}$$

the auxiliary propagators can be written as

$$\tilde{U}_{0,x_1^{\pm}x_1^{\pm}} = I_{0,x_1^{\pm}} \tag{50}$$

$$\begin{aligned} \tilde{U}_{1,x_3^{\pm}x_1^{\pm}} &= F_{x_3^{\pm},x_1^{\pm}} I_{1,x_3^{\pm}x_1^{\pm}} I_{0,x_3^{\pm}} I_{0,x_1^{\pm}} \\ &= \underbrace{F_{x_3^{\pm}x_1^{\pm}} I_{1,x_3^{\pm}x_1^{\pm}} I_{0,x_3^{\pm}}}_{M_{1,x_3^{\pm}x_1^{\pm}}} \tilde{U}_{0,x_1^{\pm}x_1^{\pm}} \end{aligned} \tag{51}$$

$$\begin{aligned} \tilde{U}_{2,x_5^{\pm}x_1^{\pm}} &= \sum_{x_3^{\pm}} F_{x_5^{\pm}x_3^{\pm}} F_{x_3^{\pm}x_1^{\pm}} I_{2,x_5^{\pm}x_1^{\pm}} I_{1,x_5^{\pm}x_3^{\pm}} I_{1,x_3^{\pm}x_1^{\pm}} I_{0,x_5^{\pm}} I_{0,x_3^{\pm}} I_{0,x_1^{\pm}} \\ &= \sum_{x_3^{\pm}} \underbrace{F_{x_5^{\pm}x_3^{\pm}} I_{1,x_5^{\pm}x_3^{\pm}} I_{0,x_5^{\pm}}}_{M_{1,x_5^{\pm}x_3^{\pm}}} \underbrace{F_{x_3^{\pm}x_1^{\pm}} I_{1,x_3^{\pm}x_1^{\pm}} I_{0,x_3^{\pm}} I_{0,x_1^{\pm}}}_{\tilde{U}_{1,x_3^{\pm}x_1^{\pm}}} \\ &\quad + (I_{2,x_5^{\pm}x_1^{\pm}} - 1) \sum_{x_3^{\pm}} F_{x_5^{\pm}x_3^{\pm}} F_{x_3^{\pm}x_1^{\pm}} I_{1,x_5^{\pm}x_3^{\pm}} I_{1,x_3^{\pm}x_1^{\pm}} I_{0,x_5^{\pm}} I_{0,x_3^{\pm}} I_{0,x_1^{\pm}}, \end{aligned} \tag{52}$$

where the underbraced M terms and the last line is analogous to the memory matrix in small matrix path integral (SMatPI) methods[19–21] (as well as the memory equation approach by Pechukas and co-workers[22]) and the transfer tensors in the transfer tensor method (TTM)[10]. While we do not use the memory matrix directly in our analysis, we note the form to connect with other known approaches.

We briefly comment on how our decomposition may be related to other known numerical approaches, such as quasiadiabatic propagator path integral (QUAPI), SMatPI, and TTM. While we emphasize that our analysis is primarily not a numerical tool to perform open quantum system dynamics, one may still wonder how it is connected to other known numerical techniques. Here, we summarize the key differences:

1. *QUAPI*: As explained in our main text, QUAPI assumes $I_k = 1$ for $k > k_{\max}$. This assumption alone does not immediately reveal $\mathbf{U}$ in terms of I_k. If one wrote $\mathbf{U}$ in terms of I_k using QUAPI without truncation, one would recover the same form of SMatPI without truncation (vide infra).
2. *SMatPI*: The SmatPI decomposition writes $\mathbf{U}$ in terms of influence functions and the bare system propagator. However, in doing so, it loses the time-translational symmetry of the memory functions, which makes the subsequent analysis we perform in this manuscript (see Section Supplementary Note 2 and Section Supplementary Note 3 F) inaccessible. Furthermore, the SMatPI approach was never formulated for problems in *Classes* 2–4.
3. *TTM*: The TTM decomposition, a black-box, data-driven, extrapolative technique, requires projection-free "numerical" inputs. In our analysis, we decompose the reduced system dynamical map in terms of the reduced system bare propagator and influence functions. This major insight is not present in TTM.

These differences make our analysis and insight presented in this manuscript unique contributions in this field.

D. Memory kernel in terms of influence functions

As alluded to in Section Supplementary Note 2, one can make appropriate substitutions to write $\mathbf{U}$ in terms of $\mathbf{I}$ and thereby write $\mathbf{K}$ in terms of $\mathbf{I}$. The direct and explicit relationship between $\mathbf{K}$ and $\mathbf{I}$, to our knowledge, has not been explicitly written for any *Classes* beyond numerical approaches using INFPI to obtain projection-free inputs for the NZ equation. We here focus on how to show this for *Class 1*.

For *Class 1*, we unravel Eq. (19) and obtain

$$(\boldsymbol{\mathcal{K}}_0)_{ik} = \frac{1}{\Delta t^2}\Big[\sum_j G_{ij} I_j^0 G_{jk} + (\frac{i}{\hbar}\mathcal{L}_s \Delta t - 1)_{ik}\Big]. \tag{53}$$

Next, from Eq. (24),

$$(\boldsymbol{\mathcal{K}}_1)_{im} = \frac{1}{\Delta t^2}\Big[\sum_j G_{ij} \sum_k F_{jk} I_{1,jk} I_{0,j} I_{0,k} G_{km} - \sum_j G_{ij} I_{0,j} \sum_k G_{jk} \sum_l G_{kl} I_{0,l} G_{lm}\Big]. \tag{54}$$

Similarly by making appropriate substitutions into $\mathbf{U}_1$, $\mathbf{U}_2$, and $\mathbf{U}_3$ in Eq. (26),

$$\begin{aligned}(\boldsymbol{\mathcal{K}}_2)_{ip} &= \frac{1}{\Delta t^2}\Big[-\sum_j G_{ij} I_{0,j} \sum_k F_{jk} I_{1,jk} I_{0,k} \sum_n F_{kn} I_{0,n} G_{np} + \sum_j G_{ij} I_{0,j} \sum_k F_{jk} I_{0,k} \sum_n F_{kn} I_{0,n} G_{np} \\ &\quad - \sum_j G_{ij} I_{0,j} \sum_k F_{jk} \sum_n F_{kn} I_{1,kn} I_{0,k} I_{0,n} G_{np} + \sum_{jkn} G_{ij} F_{jk} F_{kn} I_{2,jn} I_{1,jk} I_{1,kn} I_{0,j} I_{0,k} I_{0,n} G_{np}.\end{aligned} \tag{55}$$

We again factorize as follows

$$I_{2,jn} I_{1,jk} I_{1,kn} = (I_{2,jn} - 1) I_{1,jk} I_{1,kn} + \underbrace{I_{1,jk} + I_{1,kn} - 1} + (I_{1,jk} - 1)(I_{1,kn} - 1). \tag{56}$$

The underbraced terms cancel with the first three terms in Eq. (55) and we are left with

$$(\boldsymbol{\mathcal{K}}_2)_{ip} = \sum_{jkn} G_{ij} F_{jk} F_{kn} [(I_{2,jn} - 1) I_{1,jk} I_{1,kn} + (I_{1,jk} - 1)(I_{1,kn} - 1)] I_{0,j} I_{0,k} I_{0,n} G_{np}. \tag{57}$$

One can write down higher-order terms similarly. By making substitutions to Eq. (28),

$$\begin{aligned}(\boldsymbol{\mathcal{K}}_3)_{il} &= \sum_{jknp} G_{ij} F_{jk} F_{kn} F_{np} [I_{3,jp} I_{2,jn} I_{2,kp} I_{1,jk} I_{1,kn} I_{1,np} + I_{1,jk} - 1 + I_{1,kn} - I_{2,jn} I_{1,jk} I_{1,kn} \\ &\quad - I_{1,jk} I_{1,np} + I_{1,np} - I_{2,kp} I_{1,kn} I_{1,np}] I_{0,j} I_{0,k} I_{0,n} I_{0,p} G_{pl}.\end{aligned} \tag{58}$$

We factorize as follows

$$I_{3,jp} I_{2,jn} I_{2,kp} I_{1,jk} I_{1,kn} I_{1,np} = (I_{3,jp} - 1) I_{2,jn} I_{2,kp} I_{1,jk} I_{1,kn} I_{1,np} + I_{2,jn} I_{2,kp} I_{1,jk} I_{1,kn} I_{1,np}. \tag{59}$$

For now notice that

$$I_{2,jn} I_{2,kp} I_{1,jk} I_{1,kn} I_{1,np} - I_{2,jn} I_{1,jk} I_{1,kn} - I_{2,kp} I_{1,kn} I_{1,np} = I_{1,kn}(I_{2,jn} I_{2,kp} I_{1,jk} I_{1,np} - I_{2,jn} I_{1,jk} - I_{2,kp} I_{1,np})$$

$$= I_{1,kn}[(I_{2,jn}-1)(I_{2,kp}-1)I_{1,jk}I_{1,np} + I_{1,jk}I_{1,np} - (I_{2,jn}-1)I_{1,jk} - I_{1,jk} - (I_{2,kp}-1)I_{1,np} - I_{1,np}) \\ (I_{2,kp}-1)I_{1,jk}I_{1,np} + (I_{2,jn}-1)I_{1,jk}I_{1,np}] \quad (60)$$

because

$$\begin{aligned} I_{2,jn}I_{2,kp}I_{1,jk}I_{1,np} &= I_{2,kp}I_{1,jk}I_{1,np} + (I_{2,jn}-1)I_{2,kp}I_{1,jk}I_{1,np} \\ &= (I_{2,kp}-1)I_{1,jk}I_{1,np} + (I_{2,jn}-1)I_{1,jk}I_{1,np} + I_{1,jk}I_{1,np} \\ &\quad +(I_{2,jn}-1)I_{2,kp}I_{1,jk}I_{1,np} + (I_{2,jn}-1)(I_{2,kp}-1)I_{1,jk}I_{1,np} \end{aligned} \quad (61)$$

note there

$$\begin{aligned} & (I_{2,jn}-1)(I_{2,kp}-1)I_{1,jk}I_{1,np} + (I_{2,kp}-1)I_{1,jk}I_{1,np} + (I_{2,jn}-1)I_{1,jk}I_{1,np} - (I_{2,jn}-1)I_{1,jk} - (I_{2,kp}-1)I_{1,np} \\ = \; & (I_{2,jn}-1)(I_{2,kp}-1)I_{1,jk}I_{1,np} + (I_{2,kp}-1)(I_{1,jk}-1)I_{1,np} + (I_{2,jn}-1)(I_{1,np}-1)I_{1,jk} \end{aligned} \quad (62)$$

and also that

$$I_{1,kn}[I_{1,jk}I_{1,np} - I_{1,jk} - I_{1,np}] + I_{1,jk} - 1 + I_{1,kn} - I_{1,jk}I_{1,np} + I_{1,np} = (I_{1,jk}-1)(I_{1,kn}-1)(I_{1,np}-1). \quad (63)$$

These resulting identities then group terms in Eq. (58) to give Eq. (10) in the main text.

Now for $\mathcal{K}_4$ (we use $\tilde{I}_{i,jk} = (I_{i,jk}-1)$):

$$\begin{aligned} \mathcal{K}_{4,im} = & \frac{1}{\Delta t^2}\sum_{jknpl} G_{ij}F_{jk}F_{kn}F_{np}F_{pl}I_{0,j}I_{0,k}I_{0,n}I_{0,p}I_{0,l}G_{lm}\Big\{ \\ & \tilde{I}_{4,jl}I_{1,jk}I_{2,jn}I_{3,jp}I_{1,kn}I_{2,kp}I_{3,kl}I_{1,np}I_{2,nl}I_{1,pl} \\ & +\tilde{I}_{3,jp}I_{1,jk}I_{2,jn}I_{1,kn}I_{2,kp}I_{1,np}\tilde{I}_{3,kl}I_{2,nl}I_{1,pl} + \tilde{I}_{3,jp}I_{1,jk}I_{2,jn}I_{1,kn}I_{2,kp}I_{1,np}\tilde{I}_{2,nl}I_{1,pl} \\ & +\tilde{I}_{3,jp}I_{1,jk}I_{2,jn}I_{1,kn}I_{2,kp}I_{1,np}\tilde{I}_{1,pl} + \tilde{I}_{2,jn}I_{1,jk}I_{1,kn}\tilde{I}_{3,kl}I_{2,kp}I_{1,np}I_{2,nl}I_{1,pl} + \tilde{I}_{2,jn}I_{1,jk}I_{1,kn}\tilde{I}_{2,kp}I_{1,np}\tilde{I}_{2,nl}I_{1,pl} \\ & +\tilde{I}_{2,jn}I_{1,jk}I_{1,kn}\tilde{I}_{2,kp}I_{1,np}\tilde{I}_{1,pl} + \tilde{I}_{2,jn}I_{1,jk}I_{1,kn}\tilde{I}_{2,nl}I_{1,np}I_{1,pl} + \tilde{I}_{2,jn}I_{1,jk}I_{1,kn}\tilde{I}_{1,np}\tilde{I}_{1,pl} \\ & +\tilde{I}_{1,jk}\tilde{I}_{3,kl}I_{1,kn}I_{2,kp}I_{1,np}I_{2,nl}I_{1,pl} + \tilde{I}_{1,jk}\tilde{I}_{2,kp}I_{1,kn}I_{1,np}\tilde{I}_{2,nl}I_{1,pl} + \tilde{I}_{1,jk}\tilde{I}_{2,kp}I_{1,kn}I_{1,np}\tilde{I}_{1,pl} \\ & +\tilde{I}_{1,jk}\tilde{I}_{1,kn}\tilde{I}_{2,nl}I_{1,np}I_{1,pl} + \tilde{I}_{1,jk}\tilde{I}_{1,kn}\tilde{I}_{1,np}\tilde{I}_{1,pl}\Big\}. \end{aligned} \quad (64)$$

The number of terms grows combinatorially with the number of timesteps N. In the next section, we devise a general scheme to build memory kernels at arbitrary time $N\Delta t$ based on Dyck diagrams.

E. Nakajima-Zwanzig Dyck-Diagrammatic Formalism

The structure of terms in $\mathcal{K}_N$ via the decomposition through $\{\mathbf{I}_k\}$ has the following properties. The number of terms in $\mathcal{K}_N$ is given by Catalan's numbers[23,24] $C_N = \frac{1}{N+1}\binom{2N}{N}$, as was pointed out in Ref. 25. More importantly, the terms in $\mathcal{K}_N$ are given by Dyck path diagrams[23,24] (of which there are C_N paths). A Dyck path is a sequence of up-steps and down-steps that start and end on the x-axis but never go below. The order of a Dyck path is given by the number of up-steps, which must equal the number of down-steps. The study of Dyck paths is at the root of combinatorial mathematics[26], and algorithms already exist to generate all Dyck paths of any order.[27,28] Alternatively, one can also generate all $P^R(N,k) = k^N$ (sometimes also called k-tuples) possible permutations (repetitions allowed, since here $k=2$ accounting for up or down steps we then have $P^R(2,2)=4, P^R(4,2)=16, P^R(6,2)=64$, and so on) of $\nearrow$ and $\searrow$ and then filter (i.e., such path must have equal amounts of $\nearrow$ and $\searrow$ and at any point the path must not go below the x-axis and so on) for an order $2N$ Dyck path. To obtain the terms in $\mathcal{K}_N$ from a particular Dyck path, we map a Dyck path into an influence functional diagram based on the following rules:

1. The Dyck path is made dashed.
2. Now, one draws all possible triangles in each segment of a Dyck path, but none could be as tall as any dashed peak. This step is drawn in solid. A segment of a Dyck path is defined as the sub-path of a Dyck path which starts and ends at $x=0$ (the full path itself is not counted). A peak is when an up-step meets a down-step in that order.
3. If any dashed down path sequence does not reach $x=0$, one continues to draw it until it does, with dashed lines.
4. One rounds the vertices.

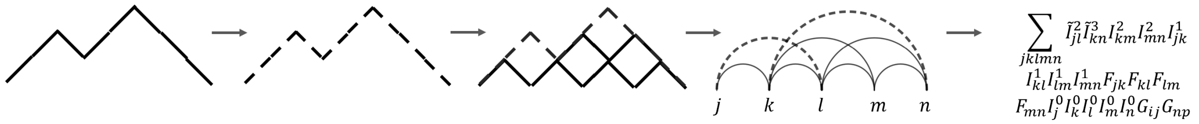

FIG. S1. Visual schematic of the algorithm to convert Dyck diagrams into terms in $\mathcal{K}$.

5. Dashed correlations connecting points i and j are $I_{ij}-1$, while solid correlations are I_{ij}. Further terms can be read immediately from the diagrams.

An example of this algorithm is shown in Fig. (S1).

We furthermore enumerate some properties of Dyck paths, which therefore unveils the general properties of $\mathcal{K}_N$:

1. The set of all Dyck paths of order n has Narayana's number, $Nr(n,k)$, the number of paths with k peaks. The number of peaks is the multiplicity of $\tilde{I}$ in the term. $Nr(n,k)$ is given by
$$Nr(n,k) = \frac{1}{n}\binom{n}{k}\binom{n}{k-1} \tag{65}$$
It is not difficult to show that $\sum_k Nr(n,k) = C_n$.
2. The number of Dyck paths in the set of all Dyck paths of order n having k number of segments is given by Catalan's triangle $C(n,k)$:
$$C(n,k) = \frac{n-k+1}{n+1}\binom{n+k}{k}. \tag{66}$$
In particular, if $n=k$, $C(n,n)$ is the nth Catalan's number C_n.
3. The number of Dyck paths in the set of all Dyck paths of order n having no hills (a segment of height 1), that is, those with no $\tilde{I}_1$ term, is given by the Fine numbers F_n with generating function $F(x)$[29]:
$$F(x) = \sum_{n\geq 0} F_n x^n = \frac{1-\sqrt{1-4x}}{x(3-\sqrt{1-4x})}. \tag{67}$$
This distribution also describes (1) Dyck paths where the leftmost peak is of even height,[29] and (2) the number of even returns to the horizontal axis (valley of height 0)[29].
4. The number of Dyck paths in the set of all Dyck paths of order $2n+2m$ with m returns to the horizontal axis is given by the Ballot numbers $B(n,m)$
$$B(n,m) = \frac{m-n}{m+n}\binom{m+n}{n}, \quad (m,n)\neq(0,0) \tag{68}$$
5. The number of Dyck paths in the set of all Dyck paths of order n with k occurrences of different $\nearrow\searrow\nearrow$ is given by $T(n,k)$, where $T(n,k)$ is related to Motzkin's number $M(k)$ via[30]
$$T(n+1,k) = \binom{n}{k}M(n-k). \tag{69}$$
Particularly, the generator of Motzkin's number $M(k)$, $m(x) = \sum_0^\infty M(n)x^n$, satisfies
$$x^2 m(x)^2 + (x-1)m(x) + 1 = 0. \tag{70}$$
6. The number of Dyck paths in the set of all Dyck paths of order n whose peaks' height total to k can be read off from Triangle A094449.[31]

Further properties of the Dyck paths have been enumerated in great detail in the On-Line Encyclopedia of Integer Sequences[31]. To construct the memory kernels via this approach, we can represent Dyck paths as Dyck Words. $\nearrow$ becomes 1 and $\searrow$ becomes 0. Hence, the Dyck path in Fig. (S1) is 11011000. By representing each path by a bit string, we can write an efficient code that enumerates all possible Dyck paths of order N.

F. Details on Computing $J(\omega)$ from $\rho(t)$

For Gaussian environments (here, we concretely consider spin-boson, but extensions to other environments are straightforward; as we discuss further in the next Supplementary Notes, we provide more details on the inverse extraction procedure that goes from reduced system dynamics, $\rho(t)$, to the bath spectral density, $J(\omega)$. We show that such a map is nearly bijective in that any aspects of $J(\omega)$ that affect the reduced system dynamics can be obtained by analyzing $\rho(t)$. This construction directly connects with Hamiltonian learning literature, as pointed out in our main text.

To proceed, we recall

$$\rho \leftrightarrow \mathbf{U} \leftrightarrow \boldsymbol{\mathcal{K}} \leftrightarrow \mathbf{I} \leftrightarrow \eta \leftrightarrow J(\omega). \tag{71}$$

We will explain step-by-step how to go from left to right. First, to obtain $\mathbf{U}_N$ from ρ_N note that $\rho_N = \mathbf{U}_N\rho_0$, where ρ_N is a vector in Liouville space with a dimension of N_L. $\mathbf{U}_N$ is then a $N_L \times N_L$ matrix. With N_L linearly-independent trajectories (obtained experimentally or computationally), we can uniquely determine $\mathbf{U}_N$. We then stack them to make the matrix

$$\mathbf{P}_N = [\rho_N^{(1)}|\rho_N^{(2)}|\dots|\rho_N^{(N_L)}]. \tag{72}$$

With this definition, we arrive at a simple linear equation to solve,

$$\mathbf{P}_N = \mathbf{U}_N\mathbf{P}_0 \tag{73}$$

By inverting $\mathbf{P}_0$, we obtain

$$\mathbf{U}_N = \mathbf{P}_N\mathbf{P}_0^{-1}. \tag{74}$$

$\mathbf{P}_0$ is invertible because each trajectory is generated from a linearly independent initial condition. While we only considered noise-free data inputs, if the data is noisy, one can use more trajectories than N_L (i.e., $\mathbf{P}_N$ and $\mathbf{P}_0$ become a fat matrix) and perform the Moore-Penrose pseudoinverse, which could help mitigate noise.

To obtain $\boldsymbol{\mathcal{K}}$ from $\mathbf{U}$ we decompose the latter iteratively, in the manner presented in the main text Eq. (6) and e.g., Ref. 10.

Next, we move to obtain $\mathbf{I}$ from $\boldsymbol{\mathcal{K}}$. First, we define $\tilde{\boldsymbol{\mathcal{K}}}_N$ which is defined through $\mathbf{G}\tilde{\boldsymbol{\mathcal{K}}}_N\mathbf{G} = \boldsymbol{\mathcal{K}}_N$. From $\tilde{\boldsymbol{\mathcal{K}}}_0$, we have

$$\Delta t^2(\tilde{\boldsymbol{\mathcal{K}}}_0 + \mathbf{G}^{-1}\mathbf{L}\mathbf{G}^{-1})_{ii} = I_{0,i} \tag{75}$$

Similarly, we have

$$\tilde{\mathcal{K}}_{1,jk} = \frac{1}{\Delta t^2} I_{0,j}F_{jk}\tilde{I}_{1,jk}I_{0,k} \;\Rightarrow\; \tilde{I}_{1,jk} = \Delta t^2 \frac{\tilde{\mathcal{K}}_{1,jk}}{F_{jk}\tilde{U}_{0,jj}\tilde{U}_{0,kk}}. \tag{76}$$

For $N = 2$, we have

$$\tilde{\mathcal{K}}_{2,jn} \;=\; \tilde{I}_{2,jn}\frac{1}{\Delta t^2}\sum_k F_{jk}F_{kn}I_{1,jk}I_{1,kn}I_{0,j}I_{0,k}I_{0,n} + \frac{1}{\Delta t^2}\sum_k F_{jk}F_{kn}\tilde{I}_{1,jk}\tilde{I}_{1,kn}I_{0,j}I_{0,k}I_{0,n}, \tag{77}$$

which leads to

$$\tilde{I}_{2,jn} \;=\; \frac{\Delta t^2\tilde{\mathcal{K}}_{2,jn} - \sum_k F_{jk}F_{kn}\tilde{I}_{1,jk}\tilde{I}_{1,kn}I_{0,j}I_{0,k}I_{0,n}}{\sum_k F_{jk}F_{kn}I_{1,jk}I_{1,kn}I_{0,j}I_{0,k}I_{0,n}}. \tag{78}$$

This procedure is, in fact, diagrammatically generalizable–its diagrammatic equation is shown in Fig. (S2). One can generate all Dyck diagrams (which can be translated into a particular term in $\boldsymbol{\mathcal{K}}_N$) of order N. From the sum of all diagrams, one moves all diagrams except the crest term to the side of $\boldsymbol{\mathcal{K}}_N$. The crest term corresponds to the diagram that contains $\tilde{\mathbf{I}}_N$. Then, one divides both sides of the equation with the rest of the terms, which then yields $\tilde{\mathbf{I}}_N$. We note the computational costs of the procedure scale (super)combinatorially with the Dyck order N as one must compute Catalan's numbers $C_N = \frac{1}{N+1}\binom{2N}{N}$ of diagrams in $\boldsymbol{\mathcal{K}}_N$, each with about $N-1$ of contractions. However, there are ample opportunities to improve this procedure numerically, for example, by including specific classes of the Dyck diagram (see Supplementary Note 3 E).

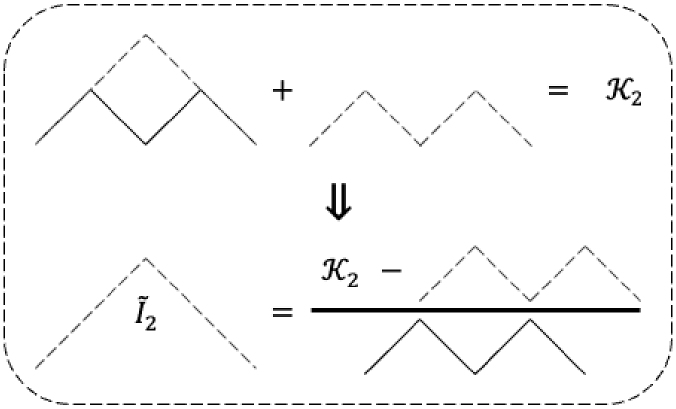

FIG. S2. Visual schematic of the inversion diagram for $\tilde{\mathbf{I}}_2$

Now to obtain η from $\mathbf{I}$, we start by taking the logarithm of Eq. (38),

$$\ln I_{k'-k,x_k^\pm x_{k'}^\pm} = -(x_k^+ - x_k^-)(\eta_{kk'} x_{k'}^+ - \eta_{kk'}^* x_{k'}^-). \tag{79}$$

Here, we consider the spin-boson model with σ_z coupling, so $x^\pm \in \text{pair}\{+1, -1\}$. In this example, we have

$$\ln I_{(k'-k)[1,-1][1,-1]} = 4\,\text{Re}\,\eta_{kk'} \tag{80}$$
$$\ln I_{(k'-k)[1,-1][1,1]} = 4i\,\text{Im}\,\eta_{kk'}. \tag{81}$$

We can also readily obtain η_{kk} from I_0 through a similar manner.

Lastly, to obtain the spectral density, $J(\omega)$, we recall that (for clarity $k - k' \to \Delta k$)

$$\eta_{\Delta k} = \int_{-\infty}^{\infty} d\omega \mathcal{F}(\omega) e^{-i\omega\Delta t\Delta k}. \tag{82}$$

where

$$\mathcal{F}(\omega) = \frac{2}{\pi}\frac{J(\omega)}{\omega^2}\frac{\exp\{\beta\hbar\omega/2\}}{\sinh\beta\hbar\omega/2}\sin^2(\omega\Delta t/2) \tag{83}$$

Note, in practice, we construct $\eta_{\Delta k}$ from $I_{kk'}$ for $\Delta k > 0$ and take the hermitian conjugate of $\eta_{\Delta k}$ for $\Delta k < 0$. We use I_{kk} for η_0. To extract $\mathcal{F}(\omega)$ with the knowledge of $\eta_{\Delta k}$ we perform a Fourier Transform

$$\mathcal{F}(\omega) = \frac{1}{2\pi}\int_{-\infty}^{\infty} dt \eta_{\Delta k} e^{i\omega\Delta t\Delta k}, \tag{84}$$

which, in practice, is performed via a discrete Fourier transform

$$\mathcal{F}(\omega) = \frac{\Delta t}{2\pi}\sum_{\Delta k=-\Delta k_{\max}}^{\Delta k_{\max}} \eta_{\Delta k} e^{i\omega\Delta t\Delta k}. \tag{85}$$

This immediately gives the spectral density through

$$J(\omega) = \mathcal{F}(\omega)\frac{\pi\omega^2\sinh\beta\hbar\omega/2}{2\sin^2(\omega\Delta t/2)\exp\{\beta\hbar\omega/2\}}. \tag{86}$$

One must pay special attention to frequencies with $\omega\Delta t/2 = n\pi$ for integer n as the denominator in Eq. (86) is zero and the expression diverges. However, this is not a problem because, physically, the spectral density at such nodal ω values does not alter the reduced system dynamics. This is because they do not contribute to $\mathcal{F}(\omega)$ as shown in Eq. (83), and can thus be seen as the null kernel of the map $J(\omega) \to \eta$. To see this, we substitute these frequencies to Eq. (85)

$$\mathcal{F}\left(\frac{2n\pi}{\Delta t}\right) = \frac{\Delta t}{2\pi}\sum_{\Delta k=-\Delta k_{\max}}^{\Delta k_{\max}} \eta_{\Delta k} e^{i2n\pi\Delta k}$$

$$= \frac{\Delta t}{2\pi} \sum_{\Delta k=-\Delta k_{\max}}^{\Delta k_{\max}} \eta_{\Delta k} \underbrace{\cos 2n\pi\Delta k + i \sin 2n\pi\Delta k}_{1}$$

$$= \frac{\Delta t}{\pi} \sum_{\Delta k=0}^{\Delta k_{\max}} \mathrm{Re}\, \eta_{\Delta k}. \tag{87}$$

However, as we substitute the nodal frequencies to Eq. (83) we then see that indeed the real part of $F(\frac{2n\pi}{\Delta t})$ also vanish identically:

$$\mathcal{F}(\frac{2n\pi}{\Delta t}) = \frac{2}{\pi} \frac{J(\omega)}{\omega^2} \frac{\exp\{\beta\hbar\omega/2\}}{\sinh \beta\hbar\omega/2} \underbrace{\sin^2 2n\pi}_{0} = 0. \tag{88}$$

Another case to consider is the pure dephasing limit where $\hat{H}_S$ is diagonal and commutes with $\hat{H}_I$. In this case, only the real part of η is available,

$$\mathrm{Re}\, \eta_{\Delta k} = \mathrm{Re} \int_{-\infty}^{\infty} d\omega \mathcal{F}(\omega) e^{-i\omega\Delta t\Delta k}$$

$$= \int_{-\infty}^{\infty} d\omega \mathcal{F}(\omega) \cos \omega\Delta t\Delta k. \tag{89}$$

We can then perform the inverse cosine transform to obtain $\mathcal{F}(\omega)$,

$$\mathcal{F}(\omega) = \frac{1}{\pi} \int_{-\infty}^{\infty} d\omega \mathrm{Re}\eta_{\Delta k} \cos \omega\Delta t\Delta k. \tag{90}$$

While the results in the main text concern sensing $J(\omega)$ of a bosonic environment, it is also possible to obtain $J(\omega)$ of a fermionic environment. For example, we employ the inversion procedure within the model in Supplementary Note 3 B 3 in Fig. S3, with the spectral density following that of Ref. 32, and find promising results.

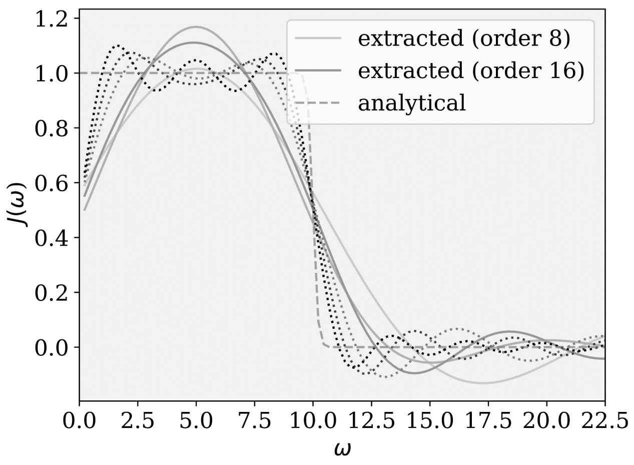

FIG. S3. Fermionic Bath spectral densities extracted through the Dyck diagrammatic method with increasing truncation orders (from white to black colors, $8, 12, 16, 20, 30$, and 40 in sequence) compared to exact spectral densities (dashed), see Supplementary Note 3 F. Note that for orders $20, 30$, and 40 (dotted), we directly proceed from $\mathbf{I} \to \eta \to J(\omega)$. Here, the fermionic spectral density is defined by defined as a flat band with a smooth edge, adopted from Ref. 33, $J(\omega) = \frac{\gamma}{(1+e^{\nu(\omega-\omega_c)})(1+e^{-\nu(\omega+\omega_c)})}$. Parameters used are: $\Delta = 1$ (other parameters are expressed relative to Δ), $\epsilon = 0$, $\beta = 50$, $\Delta t = 0.05$, $\omega_c = 10$, $\nu = 1/10$, $\mu = 0$ and $\gamma = 1$.

G. Inversion procedure with multiple environments

If we consider now a central system coupled to multiple environments (with simultaneously diagonalizable coupling operators), we will find that

$$I_{k'-k,x_k^\pm x_{k'}^\pm} \to \prod_j I^j_{k'-k,x_k^\pm x_{k'}^\pm}. \tag{91}$$

Consequently, we can perform the inversion procedure as in Supplementary Note 3 F but only up to Eq. 78. This is because what we obtain is

$$I_{k'-k,x_k^\pm x_{k'}^\pm} = \prod_j \exp\Big\{-(x^+_{j,k} - x^-_{j,k})(\eta^j_{kk'} x^+_{j,k'} - \eta^{j,*}_{kk'} x^-_{j,k'})\Big\}, \tag{92}$$

from which we cannot obtain unique values for each η. This underdetermined problem is otherwise solved if we obtain the IFs of each bath sequentially. For example, if there are two baths, we can obtain the spectral density of bath 2 from the dynamics of the system influenced by both baths 1 and 2, *if* we have access to the dynamics of the system influenced by bath 1 only.

H. Driven open quantum system dynamics

Here, we consider the explicit time dependence in the system Hamiltonian. We start by inspecting the INFPI formalism without assuming any time-translational invariance of any tensors:

$$\begin{aligned} U_{k,0,x_{2k}^\pm x_0^\pm} &= \sum_{x_{2k-1}^\pm} G_{2k-1,x_{2k}^\pm x_{2k-1}^\pm} \sum_{x_1^\pm} \tilde{U}_{k-1,0,x_{2k-1}^\pm x_1^\pm} G_{0,x_1^\pm x_0^\pm}, \\ F_{2k-1,x_{2k+1}^\pm x_{2k-1}^\pm} &= \sum_{x_{2k}^\pm} G_{2k,x_{2k+1}^\pm x_{2k}^\pm} G_{2k-1,x_{2k}^\pm x_{2k-1}^\pm}, \end{aligned} \tag{93}$$

with $G_{m,x_{m+1}^\pm x_m^\pm} = \langle x^+_{m+1}| e^{-\frac{i\hat{H}_s(t_m/2)\Delta t}{2}} |x^+_m\rangle\langle x^-_m| e^{\frac{i\hat{H}_s(t_m/2)\Delta t}{2}} |x^-_{m+1}\rangle$. There, $\hat{H}_s(t_m)$ is the system Hamiltonian at time $t_m = m\Delta t$. Hence,

$$\tilde{U}_{0,0,x_1^\pm x_1^\pm} = I_{0,x_1^\pm} \tag{94}$$

$$\begin{aligned} \tilde{U}_{1,0,x_3^\pm x_1^\pm} &= F_{1,x_3^\pm x_1^\pm} I_{1,x_3^\pm x_1^\pm} I_{0,x_3^\pm} I_{0,x_1^\pm} \\ \tilde{U}_{2,0,x_5^\pm x_1^\pm} &= \sum_{x_3^\pm} F_{3,x_5^\pm x_3^\pm} F_{1,x_3^\pm x_1^\pm} I_{2,x_5^\pm x_1^\pm} I_{1,x_5^\pm x_3^\pm} I_{1,x_3^\pm x_1^\pm} I_{0,x_5^\pm} I_{0,x_3^\pm} I_{0,x_1^\pm}. \end{aligned} \tag{95}$$

Similarly, the memory kernels are no longer time-translationally invariant. Consider

$$\boldsymbol{\mathcal{K}}_{0,0} = \mathbf{U}_{1,0} - \mathbf{L}_0 \tag{96}$$

$$\boldsymbol{\mathcal{K}}_{1,0} = \mathbf{U}_{2,0} - \mathbf{L}_1\mathbf{U}_{1,0} - \boldsymbol{\mathcal{K}}_{1,1}\mathbf{U}_{1,0} \tag{97}$$

where $\mathbf{L}_n \equiv (\mathbf{1} - \frac{i}{\hbar}\mathcal{L}_{S,n}\Delta t)$ with $\mathcal{L}_{S,n}\bullet \equiv [\hat{H}_S(n\Delta t), \bullet]$. We also have

$$\boldsymbol{\mathcal{K}}_{1,1} = \mathbf{U}_{2,1} - \mathbf{L}_1 \tag{98}$$

Hence,

$$\boldsymbol{\mathcal{K}}_{1,0} = \mathbf{U}_{2,0} - \mathbf{U}_{2,1}\mathbf{U}_{1,0}. \tag{99}$$

This allows us to write $\boldsymbol{\mathcal{K}}_{1,0}$ in terms of $\mathbf{I}$:

$$(\boldsymbol{\mathcal{K}}_{1,0})_{im} = \frac{1}{\Delta t^2}\Big[\sum_j G_{3,ij} \sum_k F_{1,jk} I_{1,jk} I_{0,j} I_{0,k} G_{0,km} - \sum_j G_{3,ij} I_{0,j} \sum_k G_{1,jk} \sum_l G_{1,kl} I_{0,l} G_{0,lm}\Big]$$

$$= \frac{1}{\Delta t^2}\Big[\sum_j G_{3,ij}\sum_k F_{1,jk}I_{1,jk}I_{0,j}I_{0,k}G_{0,km} - \sum_j G_{3,ij}I_{0,j}\sum_k F_{1,jk}I_{0,k}G_{0,km}\Big]$$

$$= \frac{1}{\Delta t^2}\Big[\sum_{jk} G_{3,ij}F_{1,jk}(I_{1,jk}-1)I_{0,j}I_{0,k}G_{0,km}\Big]. \quad (100)$$

Inspecting equations for later time $\mathcal{K}$, we observed that the structure of the $\boldsymbol{\mathcal{K}}$ remains largely unchanged except for including time indices for the bare system propagators. In particular, we collect all the terms except the time-dependent ones (only the bare system propagators) as a tensor, which is time-translational. That is,

$$\boldsymbol{\mathcal{K}}_{1+s,s;im} = \frac{1}{\Delta t^2}\Big[\sum_{jk}\underbrace{G_{s+3,ij}F_{s+1,jk}G_{s,km}}_{P^{s+2,s}_{ijkm}}T_{1,jk}\Big], \quad (101)$$

$$\boldsymbol{\mathcal{K}}_{2+s,s;ip} = \frac{1}{\Delta t^2}\sum_{jkn}\underbrace{G_{s+5,ij}F_{s+3,jk}F_{s+1,kn}G_{s,np}}_{P^{s+3,s}_{ijknp}}T_{2,jkn}, \quad (102)$$

where $T_{1,jk}=(I_{1,jk}-1)I_{0,j}I_{0,k}$, $T_{2,jkn}=(\tilde{I}_{2,jn}I_{1,jk}I_{1,kn}+\tilde{I}_{1,jk}\tilde{I}_{1,kn})I_{0,j}I_{0,k}I_{0,n}$. As mentioned in the main text, $\mathbf{T}$ takes a similar form as that of the process tensor,[34,35] but it is distinct in that it directly represents $\mathbf{K}$, as opposed to $\mathbf{U}$ as in the process tensor literature.

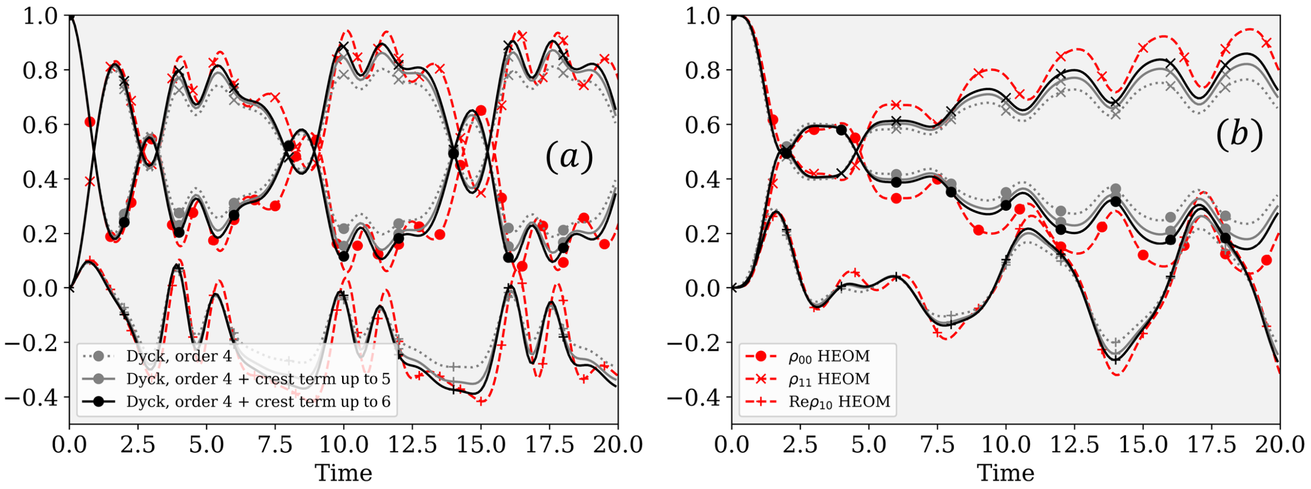

FIG. S4. Dynamics of various driven systems open to a thermal bosonic environment. Parameters used are: $\Delta=\epsilon=1$ (other parameters are expressed relative to $\Delta=\epsilon$), $\beta=5$, $\Delta t=0.1$, $\omega_c=7.5$, and $\xi=0.1$. The memory kernel is truncated at the 4th timestep for the Dyck diagrammatic method (dashed) with an additional leading term correction, the diagrams with the highest heights (smallest multiplicity), upto the sixth timestep (solid) These trajectories are compared to the exact HEOM results.

For problems with driven Hamiltonians, methods relying on time-translational invariance of memory kernels such as TTM become inapplicable to apply since the transfer tensor depends explicitly on time. Other schemes that treat this problem[21,36] seem limited to invoking periodicity and temporal symmetries, at least in terms of numerical efficiency. Here, we devise an efficient procedure to perform the reduced system dynamics, exploiting the time-translational symmetry of the Ts. The plan goes as follows: One computes all the T^i up until a predefined truncation parameter $i=r_{\max}$. Then, to obtain the kernel at time j, one performs a tensor contraction with the time-dependent bare system propagator tensor.

As a proof of concept, we consider various driven systems in Fig. (S4), where in panel (a): $\hat{H}_s=(\epsilon-\sin(t))\sigma_z+\sigma_x$, and in panel (b): $\hat{H}_s=\epsilon\sigma_z+\sin(t)\sigma_x$. In these results, we include all Dyck diagrams up to the fourth order to compute $\boldsymbol{\mathcal{K}}_0$ to $\boldsymbol{\mathcal{K}}_4$. We then approximate $\boldsymbol{\mathcal{K}}_5$ and $\boldsymbol{\mathcal{K}}_6$ by including only the diagram with the smallest multiplicity (i.e., the crest term), which is justified since the coupling here is weak and so $\tilde{I}\ll 1$. We observe that while the order 4 Dyck diagrammatic method captures the main features of the exactly computed driven dynamics (with HEOM), it is still not converged. After we include leading order correction terms up to 6 (including only the diagram with the highest height), we observe that the Dyck diagrammatic method converges to the exact values.

In panel (a), the diagonal elements of the Hamiltonian are driven. As expected, we see a lot of interlevel population crossing here. This is to be contrasted with panel (b), where the population of ρ_{11} is mostly larger than ρ_{00} except for some initial time. We also observe that for the latter, the oscillations of the populations are more regular.

Supplementary Note 4. ADDITIONAL DETAILS ON *CLASS 2*

We now start considering cases beyond our main interest, specifically beyond systems coupled to Gaussian baths where the coupling operators are all simultaneously diagonalizable. First, as an exemplifying situation we consider a system coupled to two bosonic baths through interaction Hamiltonians whose system operators do not commute, see Ref. 37 for detailed analysis. The total Hamiltonian for such a setup reads

$$H = H_S + H_{env}^a + H_{env}^b. \tag{103}$$

We may split the total Hamiltonian as

$$e^{-i\hat{H}\Delta t} \to e^{-i\hat{H}_S\Delta t} e^{-i\hat{H}^a_{\rm env}\Delta t} e^{-i\hat{H}^b_{\rm env}\Delta t} e^{-i\hat{H}_S\Delta t/2} + O(\Delta t^3). \tag{104}$$

We work in the diagonal $\hat{S}_b$ basis and insert resolution-of-the-identity in between to obtain

$$\langle b_N^+|\rho_{\rm tot}(t)|b_N^-\rangle = \langle b_N^+|e^{-i\hat{H}_S\Delta t}|b_{N-1}^+\rangle\langle b_{N-1}^+|e^{-i\hat{H}^a_{\rm env}\Delta t}e^{-i\hat{H}^b_{\rm env}\Delta t}|b_{N-2}^+\rangle\langle b_{N-2}^+|e^{-i\hat{H}_S\Delta t/2}|b_{N-3}^+\rangle\cdots. \tag{105}$$

On the left of $e^{-i\hat{H}^b_{\rm env}\Delta t}$ we insert resolution-of-the-identity in the S_a eigenbasis. The resulting term is thus

$$\begin{aligned} &\langle b_{N-1}^+|e^{-i\hat{H}^a_{\rm env}\Delta t}\,|a_N\rangle\langle a_N|\,e^{-i\hat{H}^b_{\rm env}\Delta t}|b_{N-2}^+\rangle \\ =\;& e^{-i\hat{H}^a_{\rm env}(a_N^+)\Delta t}e^{-i\hat{H}^b_{\rm env}(b_{N-2})\Delta t}\,\langle b_{N-1}^+|\,a_N\rangle\,\langle a_N|\,b_{N-2}^+\rangle \end{aligned} \tag{106}$$

we therefore have

$$\langle b_N^+|\rho(t)|b_N^-\rangle = G_{b_N^\pm b_{N-1}^\pm}G_{b_{N-1}^\pm b_{N-2}^\pm}\cdots\langle b_0^+|\rho(0)|b_0^-\rangle\,{\rm Tr}_B[e^{-i\hat{H}_{\rm env}(b_{N-1}^+)\Delta t}\cdots\rho_B\cdots e^{i\hat{H}_{\rm env}(b_{N-1}^-)\Delta t}] \tag{107}$$

where $G_{b_m^\pm b_{m-1}^\pm} = \langle b_m^+|e^{-i\hat{H}_s\Delta t/2}|b_{m-1}^+\rangle\langle b_{m-1}^-|e^{i\hat{H}_s\Delta t/2}|b_m^-\rangle$. We would then obtain that the analog to Eq. (50) is

$$\tilde{U}_{0,b_{0.33}^\pm b_{0.67}^\pm} = I^b_{0,b_{0.67}^\pm}I^a_{0,a_0^\pm}O_{b_{0.33}^\pm,a_0^\pm}O_{a_0^\pm,b_{0.67}^\pm} \tag{108}$$

$$\begin{aligned}\tilde{U}_{1,b_{1.67}^\pm b_{0.33}^\pm} =\;& I^b_{0,b_{0.67}^\pm}I^a_{0,a_0^\pm}I^b_{0,b_{1.67}^\pm}I^a_{0,a_1^\pm}I^b_{1,b_{1.67}^\pm b_{0.67}^\pm}I^a_{1,a_1^\pm a_0^\pm}O_{b_{0.33}^\pm,a_0^\pm}O_{a_0^\pm,b_{0.67}^\pm}O_{b_{1.33}^\pm,a_1^\pm}O_{a_1^\pm,b_{1.67}^\pm} \\ & G_{b_1^\pm b_{0.67}^\pm}G_{b_{0.33}^\pm b_0^\pm}\end{aligned} \tag{109}$$

$$\begin{aligned}\tilde{U}_{2,b_{2.67}^\pm b_{0.33}^\pm} =\;& I^b_{2,b_{2.67}^\pm b_{0.67}^\pm}I^a_{2,a_2^\pm a_0^\pm}I^b_{0,b_{2.67}^\pm}I^a_{0,a_2^\pm}I^b_{1,b_{2.67}^\pm b_{1.67}^\pm}I^a_{1,a_2^\pm a_1^\pm}I^b_{0,b_{0.67}^\pm}I^a_{0,a_0^\pm}I^b_{0,b_{1.67}^\pm}I^a_{0,a_1^\pm}I^b_{1,b_{1.67}^\pm b_{0.67}^\pm}I^a_{1,a_1^\pm a_0^\pm} \\ & O_{b_{0.33}^\pm,a_0^\pm}O_{a_0^\pm,b_{0.67}^\pm}O_{b_{1.33}^\pm,a_1^\pm}O_{a_1^\pm,b_{1.67}^\pm}O_{b_{2.33}^\pm,a_0^\pm}O_{a_0^\pm,b_{2.67}^\pm} \\ & G_{b_1^\pm b_{0.67}^\pm}G_{b_{0.33}^\pm b_0^\pm}G_{b_2^\pm b_{1.67}^\pm}G_{b_{1.33}^\pm b_1^\pm}\end{aligned} \tag{110}$$

with $O_{b_N^\pm a_N^\pm} = \langle b_N^\pm|\,a_N^\pm\rangle$. We can similarly decompose these propagators, for example explicitly,

$$U_{0,b_0^\pm b_1^\pm} = \sum_{b_{0.67}^\pm} G_{b_1^\pm b_{0.67}^\pm}I^b_{0,b_{0.67}^\pm}\sum_{a_0^\pm}O_{a_0^\pm,b_{0.67}^\pm}I^a_{0,a_0^\pm}\sum_{b_{0.33}^\pm}G_{b_{0.33}^\pm b_0^\pm}O_{b_{0.33}^\pm,a_0^\pm} \tag{111}$$

where the noninteger steps can be thought of as being auxiliary,

$$\begin{aligned}\tilde{U}_{1,b_{1.67}^\pm b_{0.33}^\pm} &= I^b_{0,b_{1.67}^\pm}\sum_{a_1^\pm}O_{a_1^\pm,b_{1.67}^\pm}I^a_{0,a_1^\pm}O_{b_{1.33}^\pm,a_1^\pm}\sum_{b_{0.67}^\pm}F_{b_{1.33}^\pm b_{0.67}^\pm}I^b_{0,b_{0.67}^\pm}I^b_{1,b_{1.67}^\pm b_{0.67}^\pm}\sum_{a_0^\pm}O_{b_{0.33}^\pm,a_0^\pm}I^a_{0,a_0^\pm}O_{a_0^\pm,b_{0.67}^\pm}I^a_{1,a_1^\pm a_0^\pm} \\ &= I^b_{0,b_{1.67}^\pm}\sum_{a_1^\pm}O_{a_1^\pm,b_{1.67}^\pm}I^a_{0,a_1^\pm}O_{b_{1.33}^\pm,a_1^\pm}\sum_{b_{0.67}^\pm}F_{b_{1.33}^\pm b_{0.67}^\pm}I^b_{1,b_{1.67}^\pm b_{0.67}^\pm}\tilde{U}_{0,b_{0.67}^\pm b_{0.33}^\pm}\cdots\end{aligned} \tag{112}$$

and so on. Here, it is more convenient to work with the propagators directly, and we will find

$$
\begin{aligned}
U_{2,b_2^\pm b_0^\pm} &= \sum_{b_1^\pm} U_{1,b_2^\pm b_1^\pm} U_{1,b_1^\pm b_0^\pm} \\
&+ \sum_{b_{1.67}^\pm} G_{b_2^\pm b_{1.67}^\pm} I^b_{0,b_{1.67}^\pm} \sum_{a_1^\pm} O_{a_1^\pm, b_{1.67}^\pm} I^a_{0,a_1^\pm} O_{b_{1.33}^\pm, a_1^\pm} \sum_{b_{0.67}^\pm} F_{b_{1.33}^\pm b_{0.67}^\pm} I^b_{0,b_{0.67}^\pm} (I^b_{1,b_{1.67}^\pm b_{0.67}^\pm} - 1) \\
&\quad \sum_{a_0^\pm} I^a_{0,a_0^\pm} O_{a_0^\pm, b_{0.67}^\pm} I^a_{1,a_1^\pm a_0^\pm} \sum_{b_{0.33}^\pm} O_{b_{0.33}^\pm, a_0^\pm} G_{b_{0.33}^\pm b_0^\pm} \\
&+ \sum_{b_{1.67}^\pm} G_{b_2^\pm b_{1.67}^\pm} I^b_{0,b_{1.67}^\pm} \sum_{a_1^\pm} O_{a_1^\pm, b_{1.67}^\pm} I^a_{0,a_1^\pm} O_{b_{1.33}^\pm, a_1^\pm} \sum_{b_{0.67}^\pm} F_{b_{1.33}^\pm b_{0.67}^\pm} I^b_{0,b_{0.67}^\pm} I^b_{1,b_{1.67}^\pm b_{0.67}^\pm} \\
&\quad \sum_{a_0^\pm} I^a_{0,a_0^\pm} O_{a_0^\pm, b_{0.67}^\pm} (I^a_{1,a_1^\pm a_0^\pm} - 1) \sum_{b_{0.33}^\pm} O_{b_{0.33}^\pm, a_0^\pm} G_{b_{0.33}^\pm b_0^\pm} \\
&+ \sum_{b_{1.67}^\pm} G_{b_2^\pm b_{1.67}^\pm} I^b_{0,b_{1.67}^\pm} \sum_{a_1^\pm} O_{a_1^\pm, b_{1.67}^\pm} I^a_{0,a_1^\pm} O_{b_{1.33}^\pm, a_1^\pm} \sum_{b_{0.67}^\pm} F_{b_{1.33}^\pm b_{0.67}^\pm} I^b_{0,b_{0.67}^\pm} (I^b_{1,b_{1.67}^\pm b_{0.67}^\pm} - 1) \\
&\quad \sum_{a_0^\pm} I^a_{0,a_0^\pm} O_{a_0^\pm, b_{0.67}^\pm} (I^a_{1,a_1^\pm a_0^\pm} - 1) \sum_{b_{0.33}^\pm} O_{b_{0.33}^\pm, a_0^\pm} G_{b_{0.33}^\pm b_0^\pm}.
\end{aligned} \tag{113}
$$

This equation will be compared with Eq. (22), where the second, third, and fourth terms should constitute the memory kernel. The recursive, combinatorial structure of this multi-bath problem is distinct from the one-bath/multiple additive bath problems we dealt with in *Class 1* –detailed analysis and numerical evidence in this regard are left for future work.

A. Inversion procedure for *Class 2*

For the model considered here, the inverse procedure will not follow completely the approach we outlined for *Class 1*. In particular, there is no apparent diagrammatic structure underlying the expansion of $\mathbf{U}$ in terms of $\mathbf{I}$, yet *in principle* it is possible to obtain $\mathbf{I}^\alpha$. To see this, we note that the knowledge of $\tilde{U}_{0,b_{0.33}^\pm b_{0.67}^\pm}$ (obtained tomographically) as well as the overlap matrices (otherwise we will obtain $\mathbf{I}^a$ "dressed" with the overlap terms) implies $I^b_{0,b_{0.67}^\pm} I^a_{0,a_0^\pm}$. Here, to obtain the next order $\mathbf{I}^\alpha$ we encounter the same underdetermined problem as in the multiple bath case inversion procedure in *Class 1*. So, we shall proceed here assuming the procedure is done independently-sequentially, where one of the baths' $\mathbf{I}^\alpha$ has been characterized. With this, we can thus see that we will then obtain $I^b_{1,b_{1.67}^\pm b_{0.67}^\pm} O_{a_0^\pm, b_{0.67}^\pm}$ from Eq. (109) (assuming $\mathbf{I}^a$ is the one completely characterized). Similarly, we can obtain the next order from Eq. (110).

Supplementary Note 5. ADDITIONAL DETAILS ON *CLASS 3*

We exemplify problems in this class by considering the following Hamiltonian (one bath multiple interaction Hamiltonians, so the subscript j is eliminated)

$$\hat{H} = \hat{H}_S + \hat{H}_B + \sum_\alpha \hat{S}_\alpha \otimes \hat{B}_\alpha, \tag{114}$$

where $\{\hat{B}_\alpha\}$ belong in the Hilbert space of the single bath specified by $\hat{H}_B$. For this model, let us calculate the IF, starting from Eq. (8) while here $\mathcal{L}_{I,\alpha}$ corresponds to interaction α rather than bath j there. The analog to Eq. (10) hence

$$\rho(t) = \langle \prod_\alpha \mathcal{T} e^{\int_0^t \mathcal{L}_{I,\alpha}(\tau) d\tau} \rangle \rho(0). \tag{115}$$

If one makes an independent bath approximation, the resulting multibath influence functional would miss the correlation between different coupling terms. This correlation term is what complicates *Class3* compared to *Class1*. Now, the analog to Eq. (11) is then

$$\langle \mathcal{T} e^{\int_0^t \sum_\alpha \mathcal{L}_{I,\alpha}(\tau) d\tau} \rangle = \sum_{n=0}^{\infty} \frac{1}{n!} \langle \mathcal{T} \int_0^t d\tau_1 \cdots \int_0^t d\tau_n (\sum_\alpha \mathcal{L}_{I,\alpha}(\tau_1)) \dots (\sum_\alpha \mathcal{L}_{I,\alpha}(\tau_n)) \rangle. \tag{116}$$

Here, since the sum of independent Gaussian random variables is still Gaussian, we can apply Isserlis' theorem on Eq. (116) and find that

$$\begin{aligned}\langle \mathcal{T} e^{\int_0^t \sum_\alpha \mathcal{L}_{I,\alpha}(\tau)d\tau}\rangle &= \sum_{n=0}^{\infty}\frac{1}{(2n)!}\mathcal{T}\frac{2n!}{(2^n n!)}\Big(\int_0^t d\tau_i\int_0^t d\tau_k\langle(\sum_\alpha \mathcal{L}_{I,\alpha}(\tau_i))(\sum_\alpha \mathcal{L}_{I,\alpha}(\tau_k))\rangle\Big)^n\\ &= \sum_{n=0}^{\infty}\frac{1}{n!}\Big(\int_0^t d\tau_i\int_0^t d\tau_k\frac{\langle\mathcal{T}(\sum_\alpha \mathcal{L}_{I,\alpha}(\tau_i))(\sum_\alpha \mathcal{L}_{I,\alpha}(\tau_k))\rangle}{2}\Big)^n\\ &= e^{\int_0^t d\tau\int_0^\tau d\tau'\langle\mathcal{T}(\sum_\alpha \mathcal{L}_{I,\alpha}(\tau))(\sum_\alpha \mathcal{L}_{I,\alpha}(\tau'))\rangle}\end{aligned} \tag{117}$$

in complete analogy to the derivation in Supplementary Note 1 B. Here, when discretizing Eq. (117), although one can obtain the IF, LHS of Eq. (3) in the main text, it will not factorize into pairwise separable form, the RHS of Eq. (**??**). Thus, one needs to work with the non-pairwise separable IF.

The inversion procedure is analogous to the one applied on *Class 4*, discussed in the next section. Furthermore, if one is interested to obtain the individual interaction's IF, it will not be possible to achieve this via sequential inversion in the fashion of Supplementary Note 3 G, since the correlation terms make this procedure underdetermined. Nonetheless, we can apply the technique in Supplementary Note 3 F to map this multi-interaction problem to its effective one-interaction model.

Suppose we restrict and consider a case where the system part of the interaction Hamiltonian shares a common eigenbasis and only one bath mode at a given frequency in both coupling terms. In that case, with the knowledge of the spectral density of one interaction term, we can obtain the individual interactions' spectral densities. For instance, we can obtain both spectral densities when there are two coupling terms, given access to the reduced dynamics for both couplings and one of the two couplings. This is because the correlation terms $\langle\mathcal{L}_{I,j}(\tau)\mathcal{L}_{I,k}(\tau')\rangle$ would correspond to the geometric mean spectral density, $\sqrt{J_j(\omega)}\sqrt{J_k(\omega)}$. A detailed analysis is left for future work.

Supplementary Note 6. ADDITIONAL DETAILS ON *CLASS 4*

The Feynman-Vernon Path Integral formalism employed in this work was initially developed to deal with bosonic environments, due to the simple form taken by the IF in this class of models. However, recent works have extended it to fermionic systems coupled to fermionic environments[32,38–42]. This class of problems is best exemplified by the Single Impurity Anderson Model (SIAM), whose Hamiltonian reads

$$\hat{H} = \sum_{k,\sigma} E_{k,\sigma}\hat{c}^\dagger_{k,\sigma}\hat{c}_{k,\sigma} + \sum_{k,\sigma}\Big(V_k\hat{c}^\dagger_{k,\sigma}\hat{d}_\sigma + \text{ h.c. }\Big) + U\hat{n}_\uparrow\hat{n}_\downarrow + \sum_\sigma \varepsilon_\sigma\hat{n}_\sigma. \tag{118}$$

Note that the SIAM is related to the Kondo model through the Schrieffer–Wolff transformation. The main challenges to tackle this problem with the INFPI approaches as in the main text are that (1) the coupling operators are not diagonalizable nor are they simultaneously diagonalizable. (2) the fermionic nature of *both* the impurity and the bath requires rethinking elementary operations (e.g., such as partial tracing) as the algebra is instead anticommutative.

First, with the same assumptions as in the main paper, taking the matrix elements of the total density matrix at time $t = N\Delta t$ (in the Grassmann coherent basis) we obtain

$$\langle\bar{\boldsymbol{\xi}}_t|\,\rho(t)\,|\boldsymbol{\xi}_t\rangle = \int\mathcal{D}[\bar{\boldsymbol{\xi}},\boldsymbol{\xi}]\,\langle\bar{\boldsymbol{\xi}}_{t_0}|\,\rho(t_0)\,|\boldsymbol{\xi}_{t_0}\rangle\,F[\bar{\boldsymbol{\xi}},\boldsymbol{\xi}]\prod_\sigma\mathcal{I}_\sigma\left[\bar{\boldsymbol{\xi}}_\sigma,\boldsymbol{\xi}_\sigma\right], \tag{119}$$

There, the path integral (from t_0 to t) $\mathcal{D}[\bar{\boldsymbol{\xi}},\boldsymbol{\xi}]$ term include the overcompleteness property of the Grassmann variables, $\mathbf{1} = \int d\mu(\xi)e^{-\xi^*\cdot\xi}\,|\xi\rangle\langle\xi|$. Also note that they obey Grassmann algebra $\{\xi,\hat{c}\} = 0$ and the Grassmann variables themselves also anticommute. Furthermore, the term $F[\bar{\boldsymbol{\xi}},\boldsymbol{\xi}]$ is the bare system propagator in the Grassmann coherent basis. In this representation, the form of the path integral is fundamentally different than that considered in our main work. Namely, due to the nilpotency of the Grassmann numbers, the variables ξ_n and ξ^*_n must be considered independent (and thus, compared to our main discussion these paths will be twice as long).

Regardless, one can still devise an algebraic approach to relate the memory kernel in terms of these influence functionals even in this case because they are by construction Gaussian. In particular, the IF is calculated via taking the trace of bath degrees of freedom in the Grassmann coherent basis and is given by[32,38–41] (the integration is performed via the Grassmann gaussian integral, which is equivalent to the Hubbard-Stratonovich transformation, or via the stationary path method)

$$\mathcal{I}_\sigma\left[\bar{\boldsymbol{\xi}}_\sigma,\boldsymbol{\xi}_\sigma\right] = e^{-\int_C d\tau\int_C d\tau'\bar{\xi}_\sigma(\tau)\Delta\left(\tau,\tau'\right)\xi_\sigma\left(\tau'\right)}, \tag{120}$$

with

$$\Delta\left(\tau,\tau'\right)=\int\frac{d\omega}{2\pi}J(\omega)g_{\tau,\tau'}(\omega),\quad J(\omega)=2\pi\sum_{k}\left|V_{k}\right|^{2}\delta\left(\omega-\epsilon_{k}\right),\quad g_{\tau,\tau'}(\omega)=\left(n_{\mathrm{F}}(\omega)-\Theta_{\mathcal{C}}\left(\tau,\tau'\right)\right)e^{-i\omega\left(\tau-\tau'\right)}. \tag{121}$$

Discretizing this Keldysh contour requires splitting the Grassmann variables into forward and backward branches. In the limit of a small timestep, the hybridization function takes a simple matrix form, see Refs. 38–40. Yet, to our knowledge, the discretized IF will not take a pairwise form precisely because of the Grassmann algebra. As a result, we only show the formal relationship between the IFs and the memory kernel, which is algebraic in structure. Future work will focus on finding a geometric and diagrammatic structure between the two for the SIAM.

In this model, we will find that (setting $\bar{\boldsymbol{\xi}}_j,\boldsymbol{\xi}_j\to\boldsymbol{\Xi}_j$ for brevity)

$$\begin{aligned}
\tilde{\mathbf{U}}_0[\boldsymbol{\Xi}_1] &= \mathcal{I}[\boldsymbol{\Xi}_1],\\
\tilde{\mathbf{U}}_1[\boldsymbol{\Xi}_3\boldsymbol{\Xi}_1] &= F[\boldsymbol{\Xi}_3\boldsymbol{\Xi}_1]\mathcal{I}[\boldsymbol{\Xi}_3\boldsymbol{\Xi}_1],\\
\tilde{\mathbf{U}}_2[\boldsymbol{\Xi}_5\boldsymbol{\Xi}_1] &= \sum_{\boldsymbol{\Xi}_3}F[\boldsymbol{\Xi}_5\boldsymbol{\Xi}_5]F[\boldsymbol{\Xi}_3\boldsymbol{\Xi}_1]\mathcal{I}[\boldsymbol{\Xi}_5\boldsymbol{\Xi}_3\boldsymbol{\Xi}_1],
\end{aligned} \tag{122}$$

and thus formally, for example,

$$\begin{aligned}
\mathcal{K}_1[\boldsymbol{\Xi}_4\boldsymbol{\Xi}_0] &= \frac{1}{\Delta t^2}\Big(\sum_{\boldsymbol{\Xi}_3,\boldsymbol{\Xi}_1}G[\boldsymbol{\Xi}_4\boldsymbol{\Xi}_3]F[\boldsymbol{\Xi}_3\boldsymbol{\Xi}_1]\mathcal{I}[\boldsymbol{\Xi}_3\boldsymbol{\Xi}_1]G[\boldsymbol{\Xi}_1\boldsymbol{\Xi}_0]\\
&\quad-\sum_{\boldsymbol{\Xi}_3,\boldsymbol{\Xi}_2,\boldsymbol{\Xi}_1}G[\boldsymbol{\Xi}_4\boldsymbol{\Xi}_3]\mathcal{I}[\boldsymbol{\Xi}_3]G[\boldsymbol{\Xi}_3\boldsymbol{\Xi}_2]G[\boldsymbol{\Xi}_2\boldsymbol{\Xi}_1]\mathcal{I}[\boldsymbol{\Xi}_1]G[\boldsymbol{\Xi}_1\boldsymbol{\Xi}_0]\Big),
\end{aligned} \tag{123}$$

where $\mathcal{I}[\boldsymbol{\Xi}_n\ldots\boldsymbol{\Xi}_1]$ defines the IF along the Grassmann path (note further that we also abbreviate $\boldsymbol{\xi}_n=(\boldsymbol{\xi}_{\uparrow,n},\boldsymbol{\xi}_{\downarrow,n})$) from both spin sectors.

A. Inversion procedure for the SIAM

Similarly, for the inversion procedure of problems in *Class 2*, the inverse procedure will not follow completely the approach we outlined earlier in this section. We sketch the necessary modifications here. First, we assume the density matrix has been tomographically obtained in the coherent Grassmann basis. Furthermore, despite the lack of the Dyck path structure in Eqs. 122, for the first few steps, we shall also take that we have obtained $\mathcal{K}$ in the sense of Eq. 123. Hence, by rearranging the series of equations for $\mathcal{K}_N$, we can obtain $\mathcal{I}[\boldsymbol{\Xi}_1],\mathcal{I}[\boldsymbol{\Xi}_3\boldsymbol{\Xi}_1],\mathcal{I}[\boldsymbol{\Xi}_5\boldsymbol{\Xi}_3\boldsymbol{\Xi}_1]\ldots$. Thus, recalling that $\bar{\boldsymbol{\xi}}_j,\boldsymbol{\xi}_j\to\boldsymbol{\Xi}_j$ and $\boldsymbol{\xi}_n=(\xi_{\uparrow,n},\xi_{\downarrow,n})$, by this point we have[40] (with a slight abuse of notation)

$$\begin{aligned}
\mathcal{I}_{\sigma,0} &= e^{-\sum_{\zeta,\zeta'=\pm}\bar{\xi}^{\zeta}_{\sigma,1}\Delta^{\zeta,\zeta'}_{1,1}\xi^{\zeta'}_{\sigma,1}}=1-\sum_{\zeta,\zeta'=\pm}\bar{\xi}^{\zeta}_{\sigma,1}\Delta^{\zeta,\zeta'}_{1,1}\xi^{\zeta'}_{\sigma,1},\\
\mathcal{I}_{\sigma,1} &= e^{-\sum^{2}_{j,k}\sum_{\zeta,\zeta'=\pm}\bar{\xi}^{\zeta}_{\sigma,j}\Delta^{\zeta,\zeta'}_{j,k}\xi^{\zeta'}_{\sigma,k}}=1-\sum^{2}_{j,k}\sum_{\zeta,\zeta'=\pm}\bar{\xi}^{\zeta}_{\sigma,j}\Delta^{\zeta,\zeta'}_{j,k}\xi^{\zeta'}_{\sigma,k},\\
&\;\;\vdots
\end{aligned} \tag{124}$$

where $\zeta,\zeta'=\pm$ denotes the forward-backward Keldysh branch after discretization. Now, to recollect, since we have full knowledge of the set $\mathcal{I}[\boldsymbol{\Xi}_1],\mathcal{I}[\boldsymbol{\Xi}_3\boldsymbol{\Xi}_1],\mathcal{I}[\boldsymbol{\Xi}_5\boldsymbol{\Xi}_3\boldsymbol{\Xi}_1]\ldots$ (up to a truncation order, LHS of Eqs. 124) and following, and the Grassmann numbers $\bar{\boldsymbol{\xi}}^{\zeta}_{\sigma,j},\boldsymbol{\xi}^{\zeta'}_{\sigma,j}$, we, therefore, know the hybridization matrices $\Delta^{\zeta,\zeta'}_{jk}$. This object is analogous to $\eta_{\Delta k}$ in e.g., the spin-boson case, with the difference that $\Delta^{\zeta,\zeta'}_{jk}$ takes into account the fermionic nature of the bath and the system (this comes from the $\Theta_{\mathcal{C}}$ term in Eq. (121)). Thus, one can proceed in the same fashion, continuing from Eq. (82) to obtain the fermionic spectral density from Eq. (121) by performing the appropriate Fourier Transform.

Specifically, for exemplifying purposes, suppose we have the knowledge of $\Delta\left(\tau,\tau'\right)$ (assuming we work in the Trotter limit) for all required τ,τ'. Then we define a piecewise function as follows (note that in practice this function is discretized)

$$\operatorname{Re}\Delta=\begin{cases}\operatorname{Re}\Delta\left(\tau,\tau'\right)=\operatorname{Re}\int\frac{d\omega}{2\pi}J(\omega)\left(n_{\mathrm{F}}(\omega)-1\right)e^{i\omega\left(\tau-\tau'\right)}, & \text{for } \tau-\tau'\geq 0\\ \operatorname{Re}\Delta\left(\tau',\tau\right)=\operatorname{Re}\int\frac{d\omega}{2\pi}J(\omega)\left(n_{\mathrm{F}}(\omega)-1\right)e^{-i\omega\left(\tau-\tau'\right)}, & \text{for } \tau-\tau'<0.\end{cases} \tag{125}$$

There we approximated the step function as unity at $\tau - \tau' = 0$. This piecewise function is thus continuous across $\tau - \tau'$, since the real parts of both segments are identical. So to obtain $\mathcal{F}_F(\omega) \equiv J(\omega)\,(n_{\mathrm{F}}(\omega) - 1)$ one then performs the inverse cosine transform of $\mathrm{Re}\,\Delta$, in the manner of Eqs. (89-90).

Supplementary Note 7. HEOM CALCULATIONS

All hierarchical equations of motion (HEOM)[43,44] calculations presented in this work made use of the free pole HEOM variant,[45] which makes use of the adaptive Antoulas–Anderson (AAA) algorithm[46] for constructing a simple rational function approximation to the bath noise spectrum,

$$S(\omega) = \frac{1}{2}J(\omega)\left[\coth\left(\frac{\beta\omega}{2}\right) + 1\right] \tag{126}$$

which, in turn, gives rise to a sum of exponential functions representation of the bath correlation function

$$C(t) \approx \sum_{j=1}^{K} \alpha_k e^{-\nu_k t}, \tag{127}$$

with $\mathrm{Re}(\nu_k) \geq 0$. The HEOM approach makes use of the Feynman-Vernon influence functional discussed in Supplementary Note 1, to obtain a description of the exact dynamics of an open quantum system in terms of an infinite hierarchy of auxiliary density operators (ADOs), $\hat{\rho}_{\boldsymbol{m},\boldsymbol{n}}(t)$, that encode the system-bath correlations. These ADOs are indexed by two sets of integers $\boldsymbol{m} = (m_0, m_1, \ldots, m_K)$ and $\boldsymbol{n} = (n_0, n_1, \ldots, n_K)$, each of length K.[45] This hierarchy is truncated for practical calculations such that no element of $\boldsymbol{m}$ or $\boldsymbol{n}$ is greater than some integer L.

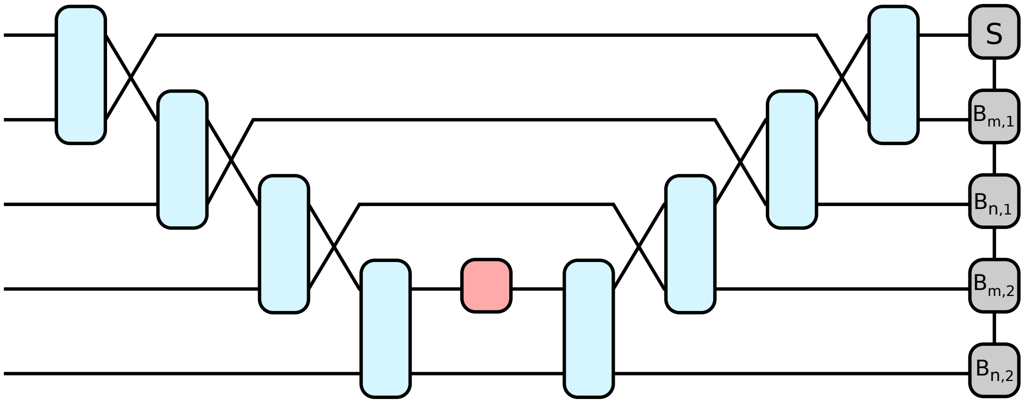

FIG. S5. An illustration of a single time step of the swap-based two-site TEBD algorithm used to integrate the FP-HEOMs. Crossing of lines indicates a swap operation applied to the MPS state. The grey rectangles represent the MPS representation of the hierarchy of ADOs, $\hat{\boldsymbol{\rho}}$. The light blue rectangles correspond to the short-time bath propagators, $\mathcal{B}_{m,k}$, $\mathcal{B}_{n,k}$, and red rectangles correspond to the short-time system propagator term, $\mathcal{U}_S(t)$.

Within the FP-HEOM method, this hierarchy of ADOs evolves according to the equations of motion[45]

$$\begin{aligned}\frac{\partial}{\partial t}\hat{\rho}_{\boldsymbol{m},\boldsymbol{n}}(t) = &-\left(i\mathcal{L}_s(t) + \sum_{k=1}^{K}(\nu_k m_k + \nu_k^* n_k)\right)\hat{\rho}_{\boldsymbol{m},\boldsymbol{n}}(t) - i\sum_{k=1}^{N}\left(\sqrt{\frac{m_k}{\|\alpha_k\|}}\alpha_k \hat{S}\hat{\rho}_{\boldsymbol{m}_k^-,\boldsymbol{n}}(t) - \sqrt{\frac{n_k}{\|\alpha_k\|}}\alpha_k^*\hat{\rho}_{\boldsymbol{m},\boldsymbol{n}_k^-}(t)\hat{S}\right) \\ &- i\sum_{k=1}^{N}\left(\sqrt{(m_k+1)\|\alpha_k\|}\left[\hat{S},\hat{\rho}_{\boldsymbol{m}_k^+,\boldsymbol{n}}(t)\right] + \sqrt{(n_k+1)\|\alpha_k\|}\left[\hat{S},\hat{\rho}_{\boldsymbol{m},\boldsymbol{n}_k^+}(t)\right]\right),\end{aligned} \tag{128}$$

where $\boldsymbol{m}_k^\pm(\boldsymbol{n}_k^\pm)$ corresponds to the set of indices $\boldsymbol{m}(\boldsymbol{n})$ but with the k-th element incremented (+) or decremented (-) by one. These equations have the general form

$$\begin{aligned}\frac{\partial}{\partial t}\hat{\boldsymbol{\rho}}(t) =& - i\mathcal{L}_s(t)\hat{\boldsymbol{\rho}}(t) + \sum_{k=1}^{N}\left[\mathcal{L}_{m,k} + \mathcal{L}_{n,k}\right]\hat{\boldsymbol{\rho}}(t) \\ =&\mathcal{M}(t)\hat{\boldsymbol{\rho}}(t),\end{aligned} \tag{129}$$

where $\mathcal{L}_{m,k}$ is an operator that acts on system and the k-th mode of the hierarchy of ADOs.

For the ohmic and subohmic spectral densities with exponential cutoffs considered in this work, the total number of exponentials in the sum of exponential representation of the bath correlation function leads to a set of auxiliary density operators that are too large to represent exactly. Following references 45, 47, and 48 we use a matrix product state (MPS) representation of the ADOs. Evolution of the HEOMs is performed through the use of a two-site time-evolving block decimation (TEBD) algorithm that makes use of a symmetric Trotter splitting of the short-time HEOM propagator,

$$\begin{aligned}\mathcal{U}_{\text{heom}}(t,t+\Delta t) &= \mathcal{T}\exp\left(\int_t^{t+\Delta t}\mathcal{M}(\tau)\mathrm{d}\tau\right)\\ &\approx \left(\prod_{k=1}^{K}\exp\left[\mathcal{L}_{m,k}\frac{\Delta t}{2}\right]\exp\left[\mathcal{L}_{n,k}\frac{\Delta t}{2}\right]\right)\exp\left[-i\mathcal{L}_S(t+\frac{\Delta t}{2})\frac{\Delta t}{2}\right]\left(\prod_{k=K}^{1}\exp\left[\mathcal{L}_{n,k}\frac{\Delta t}{2}\right]\exp\left[\mathcal{L}_{m,k}\frac{\Delta t}{2}\right]\right)\\ &= \left(\prod_{k=1}^{K}\mathcal{B}_{m,k}\mathcal{B}_{n,k}\right)\mathcal{U}_s(t)\left(\prod_{k=K}^{1}\mathcal{B}_{n,k}\mathcal{B}_{m,k}\right)\end{aligned} \tag{130}$$

In contrast to the single-site time-dependent variational principle-based schemes that have previously been used for the time evolution of tensor network-based approximations to the HEOMs,[45,47–50] the use of a two-site TEBD scheme naturally allows for adaptive control of the MPS bond dimension throughout a simulation.

The interactions present in the HEOMs given in Eq. 128 have a star topology; terms acting on two system modes all act on the system degree of freedom as well as one of the modes of the hierarchy. The application of the propagator in Eq. 130 would require the application of two-body operators acting on modes that are not nearest-neighbor in the MPS, giving rise to an algorithm that requires $O(K^2)$ two-site updates at each time step. To avoid this overhead, we employ a strategy where at the application of each two-body term, we swap the position of the system and bath site being acted upon, ensuring that at each stage the next two-body operator to apply is a local operation on the MPS.[51,52] This update scheme is illustrated schematically in Fig. S5, and requires $O(K)$ two-site updates at each time step. This scheme has the advantage that we perform a TEBD update at each stage between the system site and a single bath site. Consequentially, when the system Liouville space dimension is much smaller than the hierarchy depth, the cost of two-site updates will be dramatically reduced compared to the naive approach.

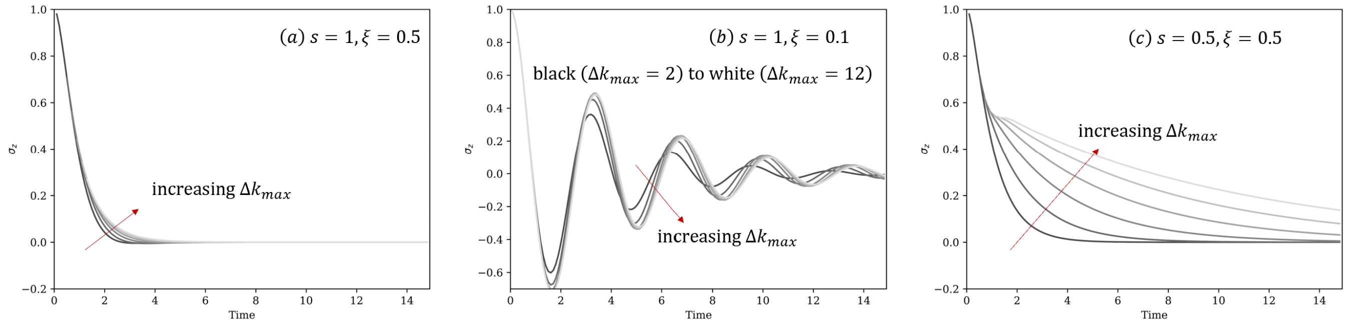

FIG. S6. Magnetization ($\langle\sigma_z(t)\rangle$) dynamics predicted using i-QuAPI method with increasing $\Delta k_{\max}$ (from black to white colors, $\Delta k_{\max}=2$ to $\Delta k_{\max}=12$.). Parameters used are: $\Delta=1$, $\epsilon=0$, $\beta=5$, $\Delta t=0.1$, $\omega_c=7.5$, and $\xi=0.1$ and $s=1$ (panel (a)), $\xi=0.5$ and $s=1$ (panel (b)), or $\xi=0.5$ and $s=0.5$ (panel (c))

Supplementary Note 8. ADDITIONAL SIMULATIONS

For Figures (2) and (3) panels (a) and (b) in the main text, the exact results from which our calculations compare come from converged HEOM results. We cross-validate these results using the i-QuAPI method, which agrees with HEOM, see Fig. (S6).

Fig. (2) panel (c) in the main text suggests that the decay of $\left\|\tilde{\mathbf{I}}_N\right\|$ (and hence $\|\boldsymbol{\mathcal{K}}_N\|$) is extremely slow. One question is if they eventually decay to zero at all. In Fig. (S7) we present $\left\|\tilde{\mathbf{I}}_N\right\|$ and $\|\boldsymbol{\mathcal{K}}_N\|$ for longer times than in the main text. We observe that neither has vanished and both are still decaying even until $t=40$.

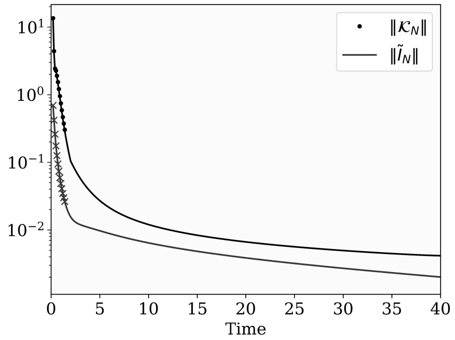

FIG. S7. Same as Fig. 2 in the main text with a longer time.

In Fig. (S8), we demonstrate the perfect agreement between inversely obtained η to the analytical η. This is the intermediate step to extract the spectral density of the environment; see Supplementary Note 3 F.

Furthermore, we show how we can inversely obtain $J(\omega)$ for highly structured environments in Fig. S9: in panel (a) $J_a(\omega) = \frac{\gamma\omega\omega_0^2}{(\omega^2-\omega_0^2)^2+\gamma^2\omega^2} + \frac{\gamma\omega\omega_0^2}{(\omega^2-4\omega_0^2)^2+\gamma^2\omega^2}$, for panel (b) $J_b(\omega) = \frac{\gamma}{(\omega-\omega_0)^2+\gamma^2} + \frac{\gamma}{(\omega-3\omega_0)^2+\gamma^2}$, and for panel (c) $J_c(\omega) = \frac{\gamma\omega\omega_0^2}{(\omega^2-\omega_0^2)^2+\gamma^2\omega^2} + \frac{\gamma}{(\omega-3\omega_0)^2+\gamma^2}$. Specifically these are spectral densities that are the additions of Brownian-Brownian, Lorentz-Lorentz, and Brownian-Lorentz spectral densities with different peak frequencies, respectively. It is significant that even for highly structured spectral densities such as these, the extraction procedure succeeds, although one would need to go to high orders in the Dyck diagrams.

Lastly, we look at how the truncation in $\boldsymbol{\mathcal{K}}$ affects $\mathbf{I}$ and vice versa. We construct approximate $\boldsymbol{\mathcal{K}}$ with truncated $\tilde{\mathbf{I}}$ (i.e., akin to i-QUAPI). Similarly, we extract effective $\mathbf{I}$ with truncated $\boldsymbol{\mathcal{K}}$ (i.e., akin to GQME). We present these results in Fig. (S10). Here, we study the three different regimes considered in the main text. In panels (a1), (b1), and (c1), we show the decay of $\|\boldsymbol{\mathcal{K}}_N\|$ of Dyck order 13, if we truncate (set $\tilde{\mathbf{I}}_{k,ij} = 0$ for $k > k_{\max}$) at $k_{\max} = 5$, $k_{\max} = 9$, and $k_{\max} = 13$ respectively. Here, one observes the error of premature truncation compounds, at e.g., $t = 1.2$ the error of $\|\boldsymbol{\mathcal{K}}_N\|$ when truncating at $k_{\max} = 5$ is significantly larger than when truncating at $k_{\max} = 9$. On the other hand, it appears that the effective $\tilde{\mathbf{I}}$ extracted from this truncated $\boldsymbol{\mathcal{K}}$ is a poor approximate of the actual $\tilde{\mathbf{I}}$ (although this makes sense since we impose a hard truncation, $\tilde{\mathbf{I}}_{k,ij} = 0$ for $k > k_{\max}$). This is shown in panels (a2), (b2), and (c2); of Dyck order 13, at $k_{\max} = 5$, $k_{\max} = 9$, and $k_{\max} = 13$, respectively.

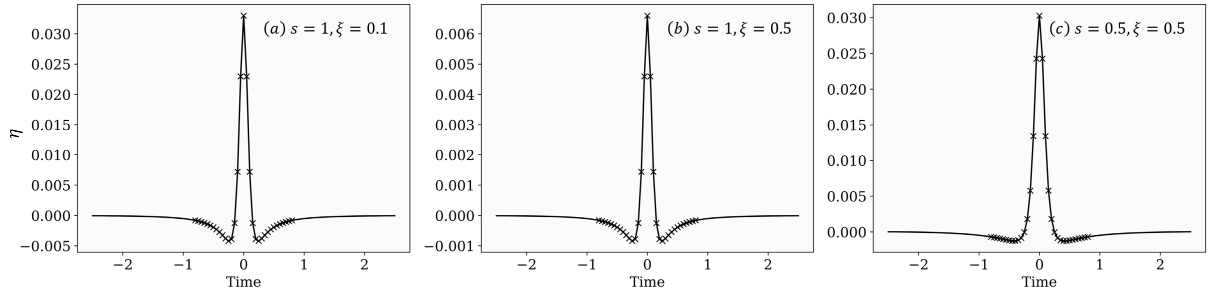

FIG. S8. The coefficients $\eta_{\Delta k}$ inversely calculated (solid line), which perfectly matches the a priori results (crosses). Parameters are identical to Fig. (3) in the main text.

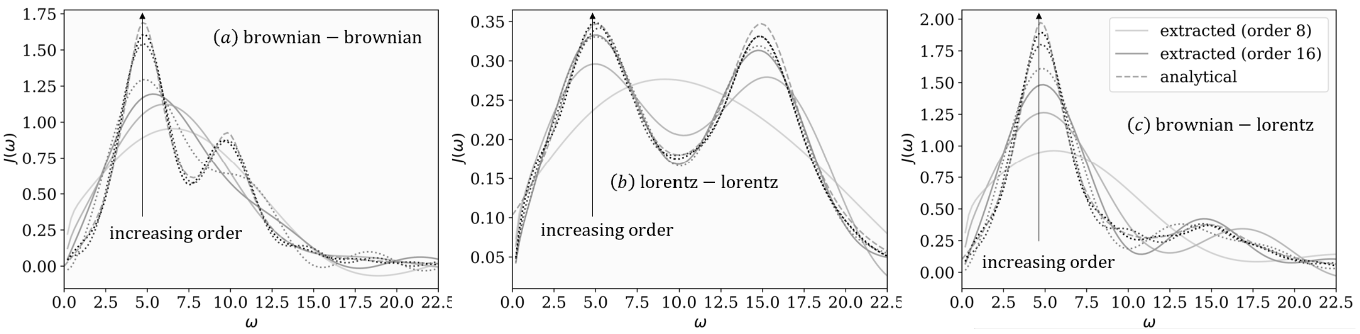

FIG. S9. Panels (a), (b), and (c): Bath spectral densities extracted through the Dyck diagrammatic method with increasing truncation orders (from white to black colors, 8, 12, 16, 20, 30, and 40 in sequence) compared to exact spectral densities (dashed), see Supplementary Note 3 F. Note that for orders 20, 30, and 40 (dotted), we directly proceed from $\mathbf{I} \to \eta \to J(\omega)$ since the procedure $\boldsymbol{\mathcal{K}} \to \mathbf{I}$ for these high orders are currently infeasible (see paragraph below Eq. (78)). In all cases, our approach obtains the structured spectral density. For panel (a) the exact spectral density $J_a(\omega) = \frac{\gamma\omega\omega_0^2}{(\omega^2-\omega_0^2)^2+\gamma^2\omega^2} + \frac{\gamma\omega\omega_0^2}{(\omega^2-4\omega_0^2)^2+\gamma^2\omega^2}$, for panel (b) $J_b(\omega) = \frac{\gamma}{(\omega-\omega_0)^2+\gamma^2} + \frac{\gamma}{(\omega-3\omega_0)^2+\gamma^2}$, and for panel (c) $J_c(\omega) = \frac{\gamma\omega\omega_0^2}{(\omega^2-\omega_0^2)^2+\gamma^2\omega^2} + \frac{\gamma}{(\omega-3\omega_0)^2+\gamma^2}$. Parameters used are: $\Delta = 1$ (other parameters are expressed relative to Δ), $\epsilon = 0$, $\beta = 5$, $\Delta t = 0.05$, $\omega_c = 7.5$, $\xi = 2$, $\gamma = \frac{\xi\pi}{2}$, and $\omega_0 = 5$.

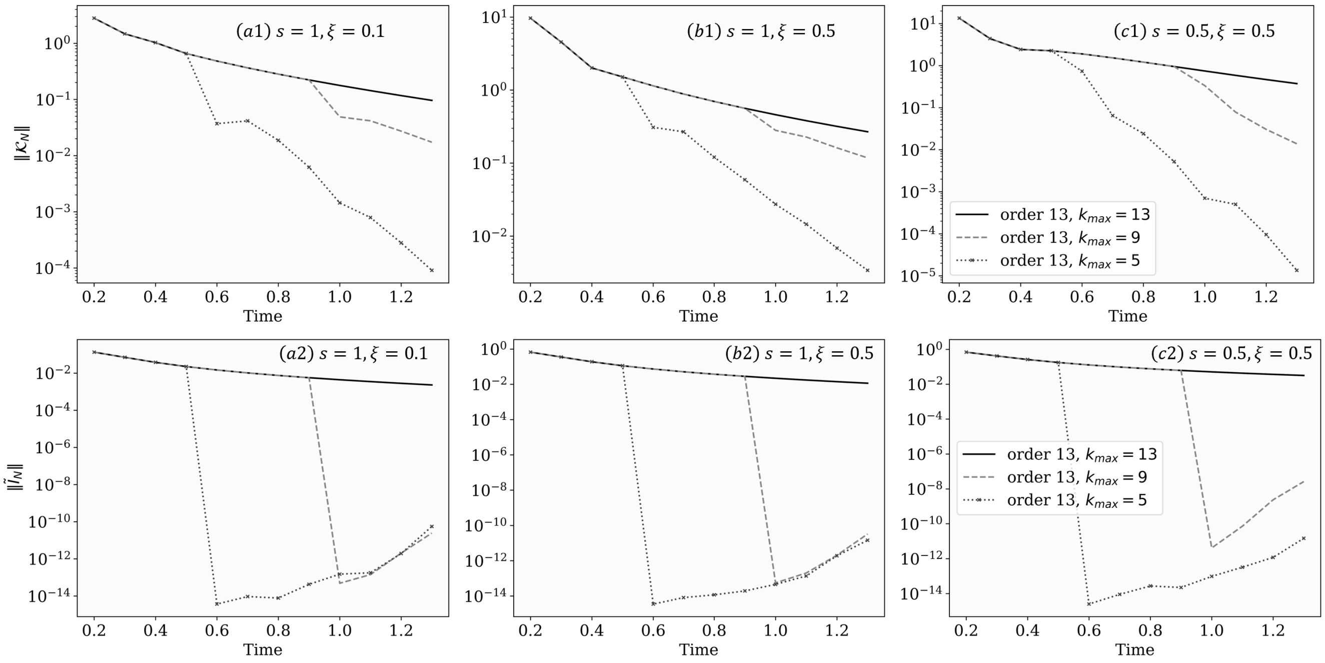

FIG. S10. Panels (a1), (b1), and (c1): The operator norm of the approximate $\boldsymbol{\mathcal{K}}_N$ when we truncate (set $\tilde{I}_{k,ij} = 0$ for $k > k_{\max}$) at $k_{\max} = 5$, $k_{\max} = 9$, and $k_{\max} = 13$, respectively. The solid line is the exact $\boldsymbol{\mathcal{K}}_N$. Panels (a2), (b2), and (c2): The operator norm of $\tilde{\mathbf{I}}_N$ obtained from approximate $\boldsymbol{\mathcal{K}}_N$ shown in panels (a1), (b1), and (c1). The solid line indicates the exact $\tilde{\mathbf{I}}_N$. Parameters used are: $\Delta = 1$ (other parameters are expressed relative to Δ), $\epsilon = 0$, $\beta = 5$, $\Delta t = 0.1$, $\omega_c = 7.5$, and $\xi = 0.1$ and $s = 1$ (panel (a)), $\xi = 0.5$ and $s = 1$ (panel (b)), or $\xi = 0.5$ and $s = 0.5$ (panel (c))

* joonholee@g.harvard.edu

[1] R. P. Feynman and F. L. Vernon, Annals of Physics **24**, 118 (1963), ISSN 0003-4916, URL https://www.sciencedirect.com/science/article/pii/000349166390068X.
[2] S. Luo, N. Lambert, P. Liang, and M. Cirio, PRX Quantum **4**, 030316 (2023), URL https://link.aps.org/doi/10.1103/PRXQuantum.4.030316.
[3] D. Gribben, A. Strathearn, G. E. Fux, P. Kirton, and B. W. Lovett, Quantum **6**, 847 (2022).

[4] D. Gribben, D. M. Rouse, J. Iles-Smith, A. Strathearn, H. Maguire, P. Kirton, A. Nazir, E. M. Gauger, and B. W. Lovett, PRX Quantum **3**, 010321 (2022), URL https://link.aps.org/doi/10.1103/PRXQuantum.3.010321.
[5] R. Kubo, Journal of the Physical Society of Japan **17**, 1100 (1962).
[6] L. Isserlis, Biometrika **12**, 134 (1918).
[7] G.-C. Wick, Physical review **80**, 268 (1950).
[8] G. Fubini, Rend. Acc. Naz. Lincei **16**, 608 (1907).
[9] S. Nakajima, Progress of Theoretical Physics **20**, 948 (1958), ISSN 0033-068X, URL https://doi.org/10.1143/PTP.20.948.
[10] J. Cerrillo and J. Cao, Phys. Rev. Lett. **112**, 110401 (2014), URL https://link.aps.org/doi/10.1103/PhysRevLett.112.110401.
[11] A. Nitzan, *Chemical Dynamics in Condensed Phases: Relaxation, Transfer, and Reactions in Condensed Molecular Systems* (New York: Oxford University Press, 2006).
[12] M. Knap, D. A. Abanin, and E. Demler, Phys. Rev. Lett. **111**, 265302 (2013).
[13] J. Jin, S. Welack, J. Luo, X.-Q. Li, P. Cui, R.-X. Xu, and Y. Yan, The Journal of chemical physics **126** (2007).
[14] K. M. Forsythe and N. Makri, Phys. Rev. B **60**, 972 (1999), URL https://link.aps.org/doi/10.1103/PhysRevB.60.972.
[15] D. Segal, The Journal of chemical physics **140** (2014).
[16] H. Wang and J. Shao, The Journal of Chemical Physics **137** (2012).
[17] V. Link, K. Luoma, and W. T. Strunz, New Journal of Physics **25**, 093006 (2023).
[18] A. O. Caldeira, A. H. Castro Neto, and T. Oliveira de Carvalho, Phys. Rev. B **48**, 13974 (1993), URL https://link.aps.org/doi/10.1103/PhysRevB.48.13974.
[19] N. Makri, Journal of Chemical Theory and Computation **17**, 1 (2021).
[20] N. Makri, The Journal of Chemical Physics **152**, 041104 (2020).
[21] S. Kundu and N. Makri, The Journal of Chemical Physics **158**, 224801 (2023), ISSN 0021-9606, https://pubs.aip.org/aip/jcp/article-pdf/doi/10.1063/5.0151748/17987394/224801_1_5.0151748.pdf, URL https://doi.org/10.1063/5.0151748.
[22] A. A. Golosov, R. A. Friesner, and P. Pechukas, The Journal of chemical physics **110**, 138 (1999).
[23] R. P. Stanley, *Catalan Numbers* (Cambridge University Press, 2015).
[24] *The on-line encyclopedia of integer sequences, sequence a000108* (2010), URL https://oeis.org/A000108.
[25] G. Wang and Z. Cai, *Tree-based implementation of the small matrix path integral for system-bath dynamics* (2022), 2207.11830.
[26] N. Vilenkin, *Combinatorics [by] N. Ya. Vilenkin* (Academic Press, 1971), URL https://books.google.com/books?id=MnNFNQAACAAJ.
[27] A. Bernini, I. Fanti, and E. Grazzini, *An exhaustive generation algorithm for catalan objects and others* (2007), math/0612127.
[28] C. Neri, *A loopless and branchless* $o(1)$ *algorithm to generate the next dyck word* (2018), 1602.06426.
[29] E. Deutsch and L. Shapiro, Discrete Mathematics **241**, 241 (2001), ISSN 0012-365X, selected Papers in honor of Helge Tverberg, URL https://www.sciencedirect.com/science/article/pii/S0012365X01001212.
[30] Y. Sun, Discrete Mathematics **287**, 177 (2004), ISSN 0012-365X, URL https://www.sciencedirect.com/science/article/pii/S0012365X04003036.
[31] OEIS Foundation Inc., *The On-Line Encyclopedia of Integer Sequences* (2023), published electronically at http://oeis.org.
[32] N. Ng, G. Park, A. J. Millis, G. K.-L. Chan, and D. R. Reichman, Physical Review B **107**, 125103 (2023).
[33] G. Cohen, E. Gull, D. R. Reichman, and A. J. Millis, Phys. Rev. Lett. **115**, 266802 (2015), URL https://link.aps.org/doi/10.1103/PhysRevLett.115.266802.
[34] M. R. Jørgensen and F. A. Pollock, Physical review letters **123**, 240602 (2019).
[35] M. R. Jørgensen and F. A. Pollock, Phys. Rev. A **102**, 052206 (2020).
[36] N. Makri, The Journal of Physical Chemistry A **125**, 10500 (2021), pMID: 34812645, https://doi.org/10.1021/acs.jpca.1c08230, URL https://doi.org/10.1021/acs.jpca.1c08230.
[37] T. Palm and P. Nalbach, The Journal of Chemical Physics **149** (2018).
[38] J. Thoenniss, M. Sonner, A. Lerose, and D. A. Abanin, Phys. Rev. B **107**, L201115 (2023), URL https://link.aps.org/doi/10.1103/PhysRevB.107.L201115.
[39] J. Thoenniss, A. Lerose, and D. A. Abanin, Physical Review B **107** (2023), ISSN 2469-9969, URL http://dx.doi.org/10.1103/PhysRevB.107.195101.
[40] R. Chen, X. Xu, and C. Guo, Physical Review B **109** (2024), ISSN 2469-9969, URL http://dx.doi.org/10.1103/PhysRevB.109.045140.
[41] J. Jin, M. W.-Y. Tu, W.-M. Zhang, and Y. Yan, New Journal of Physics **12**, 083013 (2010).
[42] M. W. Y. Tu and W.-M. Zhang, Phys. Rev. B **78**, 235311 (2008), URL https://link.aps.org/doi/10.1103/PhysRevB.78.235311.
[43] Y. Tanimura and R. Kubo, J. Phys. Soc. Jpn. **58**, 101 (1989).
[44] Y. Tanimura, J. Chem. Phys. **153** (2020), ISSN 0021-9606.
[45] M. Xu, Y. Yan, Q. Shi, J. Ankerhold, and J. T. Stockburger, Phys. Rev. Lett. **129**, 230601 (2022), URL https://link.aps.org/doi/10.1103/PhysRevLett.129.230601.
[46] Y. Nakatsukasa, O. Sète, and L. N. Trefethen, SIAM Journal on Scientific Computing **40**, A1494 (2018), https://doi.org/10.1137/16M1106122, URL https://doi.org/10.1137/16M1106122.
[47] Q. Shi, Y. Xu, Y. Yan, and M. Xu, The Journal of Chemical Physics **148**, 174102 (2018), ISSN 0021-9606, https://pubs.aip.org/aip/jcp/article-pdf/doi/10.1063/1.5026753/15539991/174102_1_online.pdf, URL https://doi.org/10.1063/1.5026753.

[48] E. Mangaud, A. Jaouadi, A. Chin, and M. Desouter-Lecomte, The European Physical Journal Special Topics **232**, 1847 (2023), ISSN 1951-6401, URL https://doi.org/10.1140/epjs/s11734-023-00919-0.

[49] L. P. J. Lindoy, D.phil. thesis, Magdalen College, University of Oxford (2019), URL https://ora.ox.ac.uk/objects/uuid:2d98ae52-9e1d-4c6d-a940-d8b9131e0275.

[50] Y. Ke, The Journal of Chemical Physics **158**, 211102 (2023), ISSN 0021-9606, https://pubs.aip.org/aip/jcp/article-pdf/doi/10.1063/5.0153870/18143581/211102_1_5.0153870.pdf, URL https://doi.org/10.1063/5.0153870.

[51] D. Bauernfeind, M. Zingl, R. Triebl, M. Aichhorn, and H. G. Evertz, Phys. Rev. X **7**, 031013 (2017), URL https://link.aps.org/doi/10.1103/PhysRevX.7.031013.

[52] D. Bauernfeind, Phd thesis, Graz University of Technology (2018), URL https://online.tugraz.at/tug_online/wbAbs.showThesis?pThesisNr=63872&pOrgNr=37.
