# Supplementary material for: Addendum: Unified framework for open quantum dynamics with memory
Source: Nat Commun. 2025 Aug 12;16:7443. doi: 10.1038/s41467-025-61825-8 (PMC12343912; doi:10.1038/s41467-025-61825-8)
Supplement: Supplementary file 1 — Supplementary Notes [file 41467_2025_61825_MOESM1_ESM.pdf]

Supplementary Notes for: Addendum to Unified framework for open quantum dynamics with memory

Ruojing Peng,[1] Felix Ivander,[2] Lachlan P. Lindoy,[3] and Joonho Lee[1]

[1] *Department of Chemistry and Chemical Biology, Harvard University, Cambridge, MA 02138, USA*

[2] *Quantum Science and Engineering, Harvard University, Cambridge, MA, USA*

[3] *National Physical Laboratory, Teddington, TW11 0LW, United Kingdom*

SUPPLEMENTARY NOTE I: DERIVATION OF EQ. (2)

In this Section, we present a derivation of Eq. (2). We start by differentiating Eq. (1) with respect to time, giving

$$\ddot{U}(t) = -iL_s\dot{U}(t) + \mathcal{K}(t) + \int_0^t d\tau \mathcal{K}(\tau)\dot{U}(t-\tau). \tag{S1}$$

Inserting Eq. (1) and rearranging gives

$$\begin{aligned}\ddot{U}(t) &= -iL_s\left[-iL_sU(t) + \int_0^t d\tau\mathcal{K}(\tau)U(t-\tau)\right] + \mathcal{K}(t)\\ &\quad + \int_0^t d\tau\mathcal{K}(\tau)\left[-iL_sU(t-\tau) + \int_0^{t-\tau} ds\mathcal{K}(s)U(t-\tau-s)\right]\\ &= (-iL_s)^2U(t) + \mathcal{K}(t) + \int_0^t d\tau\{\mathcal{K}(\tau), -iL_s\}U(t-\tau) + \int_0^t d\tau\mathcal{K}(\tau)\int_0^{t-\tau} ds\mathcal{K}(s)U(t-\tau-s).\end{aligned} \tag{S2}$$

Rearranging the double integral gives

$$\begin{aligned}\int_0^t d\tau\mathcal{K}(\tau)\int_0^{t-\tau} ds\mathcal{K}(s)U(t-\tau-s) &= \int_0^t d\tau\mathcal{K}(\tau)\int_\tau^t ds'\mathcal{K}(s'-\tau)U(t-s')\\ &= \int_0^t ds'\left[\int_0^{s'} d\tau\mathcal{K}(\tau)\mathcal{K}(s'-\tau)\right]U(t-s'),\end{aligned} \tag{S3}$$

which when used in Eq. (S2) gives Eq. (2).

SUPPLEMENTARY NOTE II: DERIVATION OF EQ. (4)

We show how the n-th order derivatives can be derived, especially for $n > 2$. If we write the n-th derivative of U in the form

$$U^{(n)} = A_nU + \int_0^t d\tau B_n(t-\tau)U(\tau) \tag{S4}$$

then

$$\begin{aligned}
U^{(n+1)} &= A_n\dot{U} + B_n(0)U + \int_0^t d\tau \frac{d}{dt}B_n(t-\tau)U(\tau) \\
&= A_n\left[-iL_sU + \int_0^t d\tau\mathcal{K}(t-\tau)U(\tau)\right] + B_n(0)U + \int_0^t d\tau\frac{d}{dt}B_n(t-\tau)U(\tau) \\
&= \left[A_n(-iL_s) + B_n(0)\right]U + \int_0^t d\tau\left[A_n\mathcal{K} + \frac{d}{dt}B_n\right](t-\tau)U(\tau) \\
&= A_{n+1}U + \int_0^t d\tau B_{n+1}(t-\tau)U(\tau) \qquad \text{(S5)}
\end{aligned}$$

where

$$A_1 = -iL_s \qquad \text{(S6)}$$

$$A_{n+1} = A_n(-iL_s) + B_n(0) \qquad \text{(S7)}$$

and

$$B_n(t) = \sum_{m=1}^{n-1} A_{n-m}\mathcal{K}^{(m-1)}(t) + \mathcal{K}^{(n-1)}(t). \qquad \text{(S8)}$$

SUPPLEMENTARY NOTE III: DERIVATION OF EQ. (14)

In this Section, we explicitly show the steps needed to arrive at Eq. (14). Starting from a Taylor series expansion for the propagator

$$U_{N+1} = U_N + \Delta t\dot{U}_N + \frac{\Delta t^2}{2}\ddot{U}_N + \frac{\Delta t^3}{6}\dddot{U}_N + \mathcal{O}(\Delta t^4), \qquad \text{(S9)}$$

we insert E1. (1), Eq. (2), and Eq. (4), giving

$$\begin{aligned}
U_{N+1} = U_N - iL_s\Delta tU_N &+ \Delta t^2\left[\frac{1}{2}\mathcal{K}_N + \sum_{m=1}^{N-1}\mathcal{K}_{N-m}U_m + \frac{1}{2}\mathcal{K}_0U_N\right] + \mathcal{O}(N\Delta t^4) \\
&+ \frac{\Delta t^2}{2}\left[(-iL_s)^2U_N + \mathcal{K}_N\right] + \frac{\Delta t^3}{2}\sum_{m=0}^{N-1}\mathcal{F}_{N-m}U_m + \mathcal{O}(N\Delta t^4) \\
&+ \frac{\Delta t^3}{6}\dddot{U}_0U_N + \frac{\Delta t^4}{6}\sum_{m=0}^{N-1}\mathcal{R}_{N-m}U_m + \mathcal{O}(N\Delta t^5). \qquad \text{(S10)}
\end{aligned}$$

Reordering terms gives Eq. (14)

$$\begin{aligned}
U_{N+1} = LU_N + \Delta t^2\sum_{m=0}^{N-1}\mathcal{K}_{N-m}U_m &+ \Delta t^2\left[\frac{1}{2}\mathcal{K}_0 + \frac{1}{2}(-iL_s)^2 + \frac{\Delta t}{6}\dddot{U}_0\right]U_N \\
&+ \frac{\Delta t^3}{2}\sum_{m=0}^{N-1}\mathcal{F}_{N-m}U_m + \mathcal{O}(N\Delta t^4) + \frac{\Delta t^4}{6}\sum_{m=0}^{N-1}\mathcal{R}_{N-m}U_m + \mathcal{O}(N\Delta t^5). \qquad \text{(S11)}
\end{aligned}$$
